# Supplementary material for: The impact of voluntary front-of-pack nutrition labelling on packaged food reformulation: A difference-in-differences analysis of the Australasian Health Star Rating scheme
Source: PLoS Med. 2020 Nov 20;17(11):e1003427. doi: 10.1371/journal.pmed.1003427 (PMC7679009; doi:10.1371/journal.pmed.1003427)

# The Impact of Voluntary Front of Pack Nutrition Labelling on Packaged Food Reformulation

## S1 Text: Descriptive Analyses

### Percent of products displaying HSR since implementation in Australia and New Zealand

Fig A: Percent Products displaying HSR labels by year in Australia and New Zealand

### Number of Observations for products that never adopted HSR, and those that adopted HSR over 2014-2018 (Aus) and 2013-2019 (NZ)a

Table A: Number of Observations for products that never adopted HSR, and those that adopted HSR over 2014-2018 (Aus) and 2013-2019 (NZ)

|  | New Zealand: Nutritrack | | | | Australia: FoodSwitch | | | |
| --- | --- | --- | --- | --- | --- | --- | --- | --- |
|  | All observations | | 2019 only | | All observations | | 2018 only | |
|  | Never HSR Labelled | Adopted HSR | Never HSR Labelled | Adopted HSR | Never HSR Labelled | Adopted HSR | Never HSR Labelled | Adopted HSR |
| Bread and bakery products | 10136 | 1463 | 1332 | 329 | 6670 | 2798 | 1348 | 812 |
|  | ( 87% ) | ( 13% ) | ( 80% ) | ( 20% ) | ( 70% ) | ( 30% ) | ( 62% ) | ( 38% ) |
| Cereal and cereal products | 7573 | 2530 | 938 | 523 | 5573 | 3055 | 856 | 896 |
|  | ( 75% ) | ( 25% ) | ( 64% ) | ( 36% ) | ( 65% ) | ( 35% ) | ( 49% ) | ( 51% ) |
| Confectionery | 5211 | 184 | 713 | 46 | 4478 | 385 | 812 | 108 |
|  | ( 97% ) | ( 3% ) | ( 94% ) | ( 6% ) | ( 92% ) | ( 8% ) | ( 88% ) | ( 12% ) |
| Convenience foods | 3591 | 1303 | 434 | 254 | 3574 | 2309 | 860 | 690 |
|  | ( 73% ) | ( 27% ) | ( 63% ) | ( 37% ) | ( 61% ) | ( 39% ) | ( 55% ) | ( 45% ) |
| Dairy | 11443 | 1618 | 1587 | 326 | 8650 | 1756 | 1818 | 478 |
|  | ( 88% ) | ( 12% ) | ( 83% ) | ( 17% ) | ( 83% ) | ( 17% ) | ( 79% ) | ( 21% ) |
| Edible oils and oil emulsions | 1804 | 249 | 254 | 46 | 1376 | 298 | 282 | 79 |
|  | ( 88% ) | ( 12% ) | ( 85% ) | ( 15% ) | ( 82% ) | ( 18% ) | ( 78% ) | ( 22% ) |
| Eggs | 504 | 79 | 71 | 16 | NA | NA | NA | NA |
|  | ( 86% ) | ( 14% ) | ( 82% ) | ( 18% ) |  |  |  |  |
| Fish and seafood products | 2671 | 562 | 300 | 110 | 1949 | 990 | 367 | 265 |
|  | ( 83% ) | ( 17% ) | ( 73% ) | ( 27% ) | ( 66% ) | ( 34% ) | ( 58% ) | ( 42% ) |
| Fruit and vegetables | 9833 | 2771 | 1262 | 559 | 6700 | 3289 | 1208 | 799 |
|  | ( 78% ) | ( 22% ) | ( 69% ) | ( 31% ) | ( 67% ) | ( 33% ) | ( 60% ) | ( 40% ) |
| Meat and meat products | 5758 | 1288 | 688 | 264 | 3701 | 1379 | 762 | 472 |
|  | ( 82% ) | ( 18% ) | ( 72% ) | ( 28% ) | ( 73% ) | ( 27% ) | (62% ) | (38%) |
| Non-alcoholic beverages | 7391 | 410 | 1020 | 114 | 5297 | 1070 | 1006 | 284 |
|  | (95% ) | (5% ) | (90% ) | (10% ) | (83% ) | (17%) | (78%) | (22%) |
| Sauces and spreads | 9956 | 2137 | 1316 | 429 | 5450 | 1214 | 1395 | 393 |
|  | (82%) | (18%) | (75%) | (25%) | (82%) | (18%) | (78%) | (22%) |
| Snackfoods | 2934 | 385 | 446 | 94 | 2108 | 468 | 451 | 151 |
|  | (88%) | (12%) | (83%) | (17%) | (82%) | (18%) | (75%) | (25%) |
| Sugars, honey and related products | 1889 | 84 | 267 | 20 | 1251 | 154 | 236 | 45 |
|  | (96%) | (4%) | (93%) | (7%) | (89%) | (11%) | (84%) | (16%) |
| Total | 80694 | 15063 | 10628 | 3130 | 56777 | 19165 | 11401 | 5472 |
|  | (84%) | (16%) | (77%) | (23%) | (75%) | (25%) | (68%) | (32%) |

### Trends in Nutrient Composition for Overall Sample across datasets: 2014-2018 (Aus) and 2013-2019 (NZ)

Fig B: Trends in nutrient composition, across the Overall Nutritrack (2013-2019) and FoodSwitch (2014-2018) samples


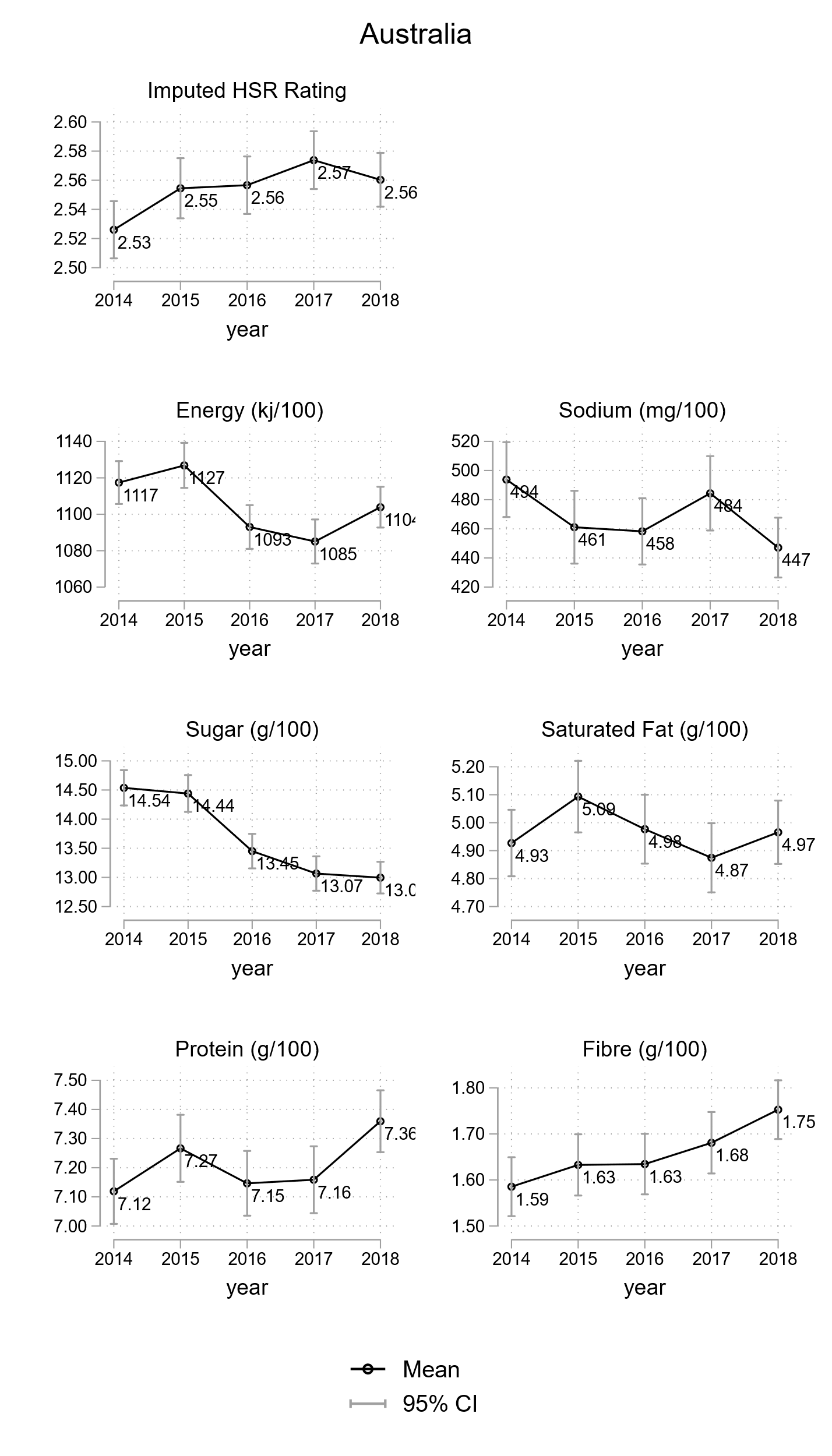

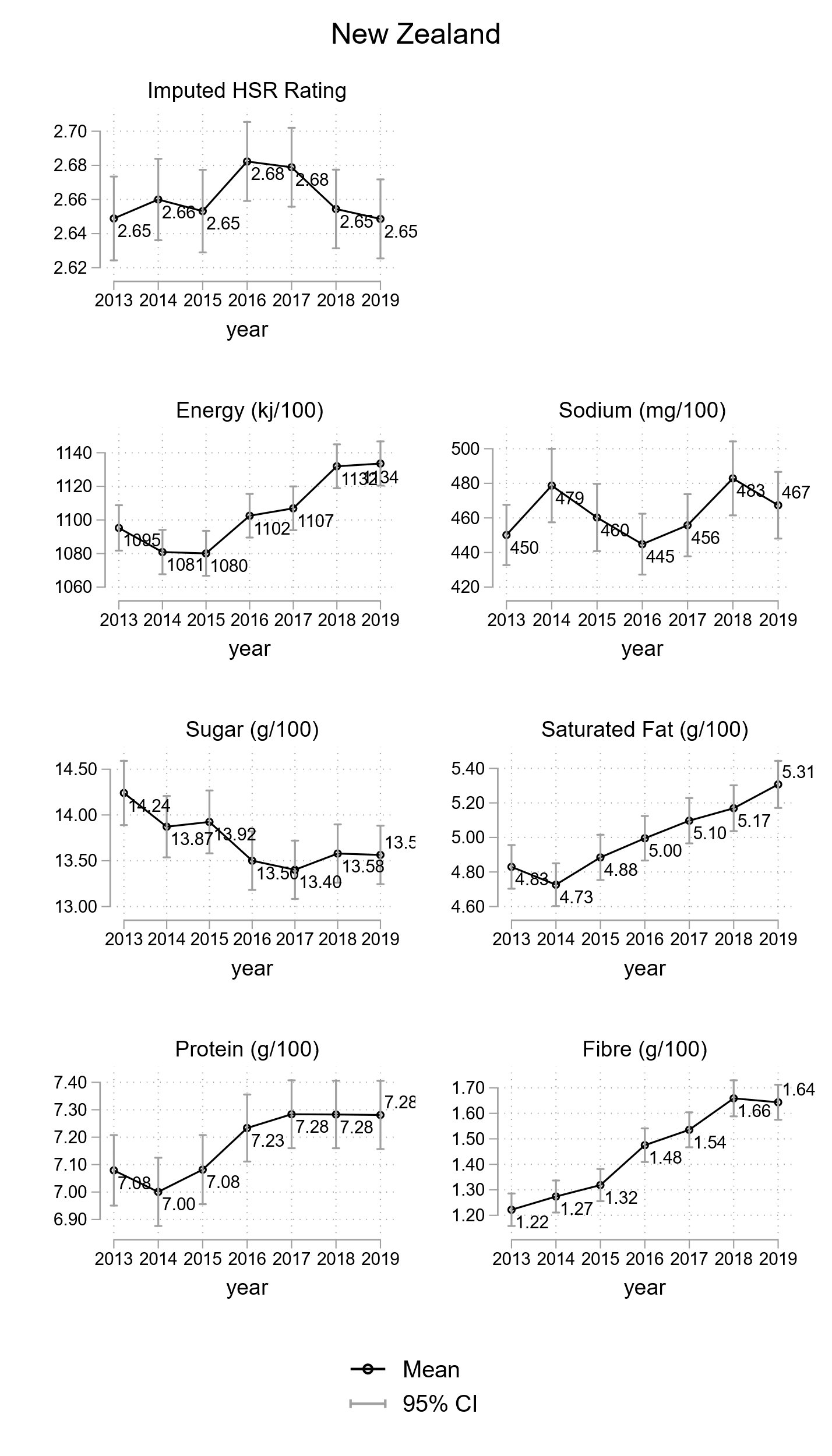

Supplement: S1 Text — (DOCX) [file pmed.1003427.s001.docx]
