# Supplementary material for: The impact of voluntary front-of-pack nutrition labelling on packaged food reformulation: A difference-in-differences analysis of the Australasian Health Star Rating scheme
Source: PLoS Med. 2020 Nov 20;17(11):e1003427. doi: 10.1371/journal.pmed.1003427 (PMC7679009; doi:10.1371/journal.pmed.1003427)
Supplement: S2 Text — (DOCX) [file pmed.1003427.s002.docx]

# The Impact of Voluntary Front of Pack Nutrition Labelling on Packaged Food Reformulation

## S2 Text: Methodological Note on reconstituted foods/“as-prepared” Nutrition Information Panels(NIPs)

This appendix discusses differences in the treatment of products that require reconstitution or preparation before consumption across datasets.

### FoodSwitch (Australia)

FoodSwitch (AU) tracks whether NIPs are reported as “As-sold” or “As-prepared” when more than one NIP is reported on the product. Only 2.0% of products reported more than one NIP. For products that report only one NIP, the NIP reported on the packet is used, but the type could not be identified by us. In cases with $\geq\boldsymbol{2}$ NIPs, the “as-prepared” NIP is used for consistency across types. The top 5 food groups with multiple NIPs are sauces (includes gravies), breakfast cereals, cake mixes, pasta, coffee & tea, and rice.

Consistent coding within FoodSwitch prevents biases in the coefficients due to the fixed effects analyses from inconsistent NIP information. Further, standard errors are smaller due to such consistent coding.

### Nutritrack (New Zealand)

A variable in the Nutritrack (NZ) dataset indicates if a product Nutrition Information Panel (NIP) reports the “as sold” product composition or the composition when reconstituted. The “as sold” composition data are the default in the database unless only reconstituted composition is reported on a product NIP. 4.6% of product NIPs report reconstituted composition.

Of these foods, most are reconstituted with water. The top 10 foods groups with reconstituted nutrition data are: Dry soup mix, flavoured noodles, coffee, gravies or stocks, cordials, yogurt dry mix, tea, pasta, and cake mixes. Thus >95% product nutrition information used in our analyses was for non-reconstituted products. Our results for NZ are largely unchanged, when removing such reconstituted foods, and the results on removing such foods are in Table 1 below:

Table A: Results for Nutritrack (New Zealand), removing reconstituted foods.

|  | HSR Rating | Energy | Sodium | Sugar | Protein | Saturated Fat | Fibre |
| --- | --- | --- | --- | --- | --- | --- | --- |
| HSR Treatment Effect | 0.07*** | -3.76+ | -18.80*** | -0.28*** | -0.04 | -0.04 | 0.04* |
|  | [0.06,0.09] | [-8.08,0.56] | [-26.81,-10.80] | [-0.40,-0.15] | [-0.10,0.02] | [-0.09,0.02] | [0.00,0.08] |
|  |  |  |  |  |  |  |  |
| Observations | 85807 | 88785 | 88329 | 88066 | 88785 | 88810 | 90621 |

95% confidence intervals in brackets

+ p < 0.10, * p < 0.05, ** p < 0.01, *** p < 0.001
