# Supplementary material for: The impact of voluntary front-of-pack nutrition labelling on packaged food reformulation: A difference-in-differences analysis of the Australasian Health Star Rating scheme
Source: PLoS Med. 2020 Nov 20;17(11):e1003427. doi: 10.1371/journal.pmed.1003427 (PMC7679009; doi:10.1371/journal.pmed.1003427)
Supplement: S3 Text — (DOCX) [file pmed.1003427.s003.docx]

# The Impact of Voluntary Front of Pack Nutrition Labelling on Packaged Food Reformulation

## S3 Text: Tables underlying Figure 3,4

Table A: Table corresponding to Figure 3,4 for Australia.

### Australia

|  | HSR Rating | Energy (KJ per 100g/ml) | Sodium (mg per 100g/ml) | Sugar (g per 100g/ml) | Protein (g per 100g/ml) | Saturated Fat (g per 100g/ml) | Fibre (g per 100g/ml) |
| --- | --- | --- | --- | --- | --- | --- | --- |
| 0.5 to 1.5 | 0.126 | -13.744 | -12.160 | -0.721 | -0.078 | -0.205 | 0.085 |
|  | [0.094,0.157] | [-22.790,-4.697] | [-27.664,3.343] | [-1.041,-0.401] | [-0.173,0.018] | [-0.352,-0.058] | [0.014,0.156] |
|  |  |  |  |  |  |  |  |
| 2.0 to 3.5 | 0.035 | -4.396 | -5.338 | 0.011 | 0.030 | 0.071 | -0.050 |
|  | [0.016,0.053] | [-8.818,0.026] | [-10.055,-0.622] | [-0.139,0.162] | [-0.022,0.083] | [0.015,0.128] | [-0.093,-0.006] |
|  |  |  |  |  |  |  |  |
| 4.0 to 5.0 | -0.035 | 0.982 | 1.569 | 0.084 | -0.027 | 0.107 | -0.088 |
|  | [-0.056,-0.014] | [-3.327,5.290] | [-3.331,6.468] | [-0.015,0.183] | [-0.118,0.064] | [0.066,0.147] | [-0.196,0.019] |
| N | 70,680 | 70,680 | 70,680 | 70,680 | 70,664 | 70,680 | 70,680 |

95% confidence intervals in brackets

Table B: Table corresponding to Figure 3,4 for New Zealand.

### New Zealand

|  | HSR Rating | Energy (KJ per 100g/ml) | Sodium (mg per 100g/ml) | Sugar (g per 100g/ml) | Protein (g per 100g/ml) | Saturated Fat (g per 100g/ml) | Fibre (g per 100g/ml) |
| --- | --- | --- | --- | --- | --- | --- | --- |
| 0.5 to 1.5 | 0.101 | -2.200 | -52.924 | -0.458 | -0.005 | -0.088 | 0.031 |
|  | [0.064,0.138] | [-13.280,8.881] | [-90.654,-15.194] | [-0.962,0.046] | [-0.149,0.139] | [-0.395,0.220] | [-0.005,0.067] |
|  |  |  |  |  |  |  |  |
| 2.0 to 3.5 | 0.112 | -4.636 | -14.374 | -0.282 | -0.029 | -0.095 | 0.064 |
|  | [0.083,0.140] | [-11.424,2.153] | [-30.293,1.544] | [-0.458,-0.106] | [-0.097,0.040] | [-0.159,-0.030] | [0.024,0.104] |
|  |  |  |  |  |  |  |  |
| 4.0 to 5.0 | 0.014 | 3.927 | -8.032 | -0.071 | -0.021 | 0.030 | 0.029 |
|  | [-0.002,0.029] | [-2.004,9.858] | [-13.922,-2.142] | [-0.201,0.060] | [-0.116,0.073] | [-0.010,0.070] | [-0.041,0.099] |
| *N* | 86210 | 87431 | 87027 | 87021 | 87431 | 87402 | 88232 |

95% confidence intervals in brackets
