# Supplementary material for: The impact of voluntary front-of-pack nutrition labelling on packaged food reformulation: A difference-in-differences analysis of the Australasian Health Star Rating scheme
Source: PLoS Med. 2020 Nov 20;17(11):e1003427. doi: 10.1371/journal.pmed.1003427 (PMC7679009; doi:10.1371/journal.pmed.1003427)
Supplement: S4 Text — (DOCX) [file pmed.1003427.s004.docx]

# The Impact of Voluntary Front of Pack Nutrition Labelling on Packaged Food Reformulation

## S4 Text: Check for Parallel Trends (Pre-existing differential trends)

### Methods

Our results can be interpreted to be the causal if the parallel paths assumption is correct, namely that the unobserved factors that cause the introduction of HSR-labelling are captured by time-invariant attributes ($\gamma_{p}$ in eq. 1), or time-varying factors that are constant amongst groups ($\mu_{t}$). Product level differences in the year of HSR labelling make it challenging to visually identify differences in trends before- and after- HSR labelling. We, therefore, also estimate the following event study regression for outcomes $y_{pt}$:

$$y_{pt}=\alpha+\boldsymbol{\gamma}_{p}+\boldsymbol{\mu}_{t}+\sum_{\tau} \beta_{\tau}\times\boldsymbol{D}_{\tau, pt}+\varepsilon_{pt} (2)$$

Where, $\tau_{tp}$ is a variable representing different time periods before and after HSR-labelling. For instance, $\tau=-2$ two years before a product gets HSR labelling, τ=0 in the year in which HSR is first displayed; $\tau=2$ is two years after HSR is implemented. τ is set to zero for all products in the comparison group. We set $\beta_{(\tau=-1)}$to be the reference group, normalised to zero, to identify differences in relative levels of the outcome before- and after- implementation of HSR, and the resulting estimates and their confidence intervals are then plotted. These estimates help to visually identify relative trends in the outcome variable, as well as the effect of the program. Further, although the parallel paths assumption is counterfactual, the lack of statistically coefficient estimates for $\tau$ before HSR-labelling increase the likelihood that the assumption holds.

### Australia

#### Stars

Fig A Pre- and post-HSR scores for foods displaying HSR labelling (indexed to zero for year of HSR being implemented), controlling for market-wide trends and time-invariant product level characteristics. Error bars are 95% Cis

#### Nutrients

Fig B: Pre- and post-HSR nutrient levels for foods displaying HSR labelling (indexed to zero for year of HSR being implemented), controlling for market-wide trends and time-invariant product level characteristics. Error bars are 95% CIs

### New Zealand

#### Stars

Fig C: Pre- and post-HSR scores for foods displaying HSR labelling (indexed to zero for year of HSR being implemented), controlling for market-wide trends and time-invariant product level characteristics. Error bars are 95% Cis

#### Nutrients

Fig D: Pre- and post-HSR nutrient levels for foods displaying HSR labelling (indexed to zero for year of HSR being implemented), controlling for market-wide trends and time-invariant product level characteristics. Error bars are 95% CIs
