# Supplementary material for: The impact of voluntary front-of-pack nutrition labelling on packaged food reformulation: A difference-in-differences analysis of the Australasian Health Star Rating scheme
Source: PLoS Med. 2020 Nov 20;17(11):e1003427. doi: 10.1371/journal.pmed.1003427 (PMC7679009; doi:10.1371/journal.pmed.1003427)
Supplement: S5 Text — (DOCX) [file pmed.1003427.s005.docx]

# The Impact of Voluntary Front of Pack Nutrition Labelling on Packaged Food Reformulation

## S5 Text: Robustness checks (Coarsened Exact Matching and DDD Estimates)

### Australia

Table A: Robustness checks (Coarsened Exact Matching and DDD Estimates) for FoodSwitch data on the outcome: Stars

|  | (1) | (2) | (3) | (4) | (5) |
| --- | --- | --- | --- | --- | --- |
|  | Baseline | Diff. Trend | CEM Weights: Nutrient Info Only | CEM Weights: Nutrient Info + Food Group | CEM Weights with Food Group + Trend |
| HSR Reformulation Effect | 0.031 | 0.027 | 0.039 | 0.038 | 0.028 |
|  | [0.017,0.044] | [0.012,0.043] | [0.025,0.054] | [0.023,0.054] | [0.012,0.044] |
|  |  |  |  |  |  |
| Diff. trend. |  | 0.003 |  |  | 0.007 |
|  |  | [-0.001,0.008] |  |  | [0.001,0.012] |
|  |  |  |  |  |  |
| Year FE | Yes | Yes | Yes | Yes | Yes |
| *N* | 77796 | 68756 | 53407 | 45725 | 43928 |
| Differential Trend | No | Yes | No | No | Yes |
| CEM Weights | No | No | Yes | Yes | Yes |

95% confidence intervals in brackets

Table B: Robustness checks (Coarsened Exact Matching and DDD Estimates) for FoodSwitch data on the outcome: Energy (kJ/100g or ml)

|  | (1) | (2) | (3) | (4) | (5) |
| --- | --- | --- | --- | --- | --- |
|  | Baseline | Diff. Trend | CEM Weights: Nutrient Info Only | CEM Weights: Nutrient Info + Food Group | CEM Weights with Food Group + Trend |
| HSR Reformulation Effect | -4.614 | -5.172 | -7.214 | -5.148 | -4.616 |
|  | [-7.932,-1.296] | [-9.232,-1.112] | [-10.777,-3.651] | [-8.533,-1.763] | [-8.845,-0.746] |
|  |  |  |  |  |  |
| Diff. trend. |  | -0.089 |  |  | -0.593 |
|  |  | [-1.673,1.494] |  |  | [-2.273,1.088] |
|  |  |  |  |  |  |
| Year FE | Yes | Yes | Yes | Yes | Yes |
| *N* | 78232 | 69190 | 53566 | 45856 | 44058 |
| Differential Trend | No | Yes | No | No | Yes |
| CEM Weights | No | No | Yes | Yes | Yes |

95% confidence intervals in brackets

Table C: Robustness checks (Coarsened Exact Matching and DDD Estimates) for FoodSwitch data on the outcome: Sodium (mg/100g or ml)

|  | (1) | (2) | (3) | (4) | (5) |
| --- | --- | --- | --- | --- | --- |
|  | Baseline | Diff. Trend | CEM Weights: Nutrient Info Only | CEM Weights: Nutrient Info + Food Group | CEM Weights with Food Group + Trend |
| HSR Reformulation Effect | -4.733 | -7.629 | -6.301 | -5.732 | -6.109 |
|  | [-9.356,-0.111] | [-12.162,-3.097] | [-10.184,-2.419] | [-10.168,-1.296] | [-10.274,-1.944] |
|  |  |  |  |  |  |
| Diff. trend. |  | 1.559 |  |  | 0.408 |
|  |  | [-1.028,4.145] |  |  | [-1.748,2.563] |
|  |  |  |  |  |  |
| Year FE | Yes | Yes | Yes | Yes | Yes |
| *N* | 78339 | 69296 | 53582 | 45871 | 44073 |
| Differential Trend | No | Yes | No | No | Yes |
| CEM Weights | No | No | Yes | Yes | Yes |

95% confidence intervals in brackets

Table D Robustness checks (Coarsened Exact Matching and DDD Estimates) for FoodSwitch data on the outcome: Sugar (g/100g or ml)

|  | (1) | (2) | (3) | (4) | (5) |
| --- | --- | --- | --- | --- | --- |
|  | Baseline | Diff. Trend | CEM Weights: Nutrient Info Only | CEM Weights: Nutrient Info + Food Group | CEM Weights with Food Group + Trend |
| HSR Reformulation Effect | -0.102 | -0.113 | -0.134 | -0.113 | -0.077 |
|  | [-0.209,0.005] | [-0.232,0.007] | [-0.237,-0.031] | [-0.215,-0.010] | [-0.181,0.027] |
|  |  |  |  |  |  |
| Diff. trend. |  | -0.012 |  |  | -0.033 |
|  |  | [-0.052,0.029] |  |  | [-0.065,-0.000] |
|  |  |  |  |  |  |
| Year FE | Yes | Yes | Yes | Yes | Yes |
| *N* | 78149 | 69107 | 53563 | 45853 | 44055 |
| Differential Trend | No | Yes | No | No | Yes |
| CEM Weights | No | No | Yes | Yes | Yes |

95% confidence intervals in brackets

Table E Robustness checks (Coarsened Exact Matching and DDD Estimates) for FoodSwitch data on the outcome: Protein (g/100g or ml)

|  | (1) | (2) | (3) | (4) | (5) |
| --- | --- | --- | --- | --- | --- |
|  | Baseline | Diff. Trend | CEM Weights: Nutrient Info Only | CEM Weights: Nutrient Info + Food Group | CEM Weights with Food Group + Trend |
| HSR Reformulation Effect | -0.011 | 0.011 | -0.026 | -0.036 | -0.011 |
|  | [-0.054,0.032] | [-0.052,0.074] | [-0.068,0.017] | [-0.080,0.008] | [-0.060,0.039] |
|  |  |  |  |  |  |
| Diff. trend. |  | -0.007 |  |  | -0.015 |
|  |  | [-0.036,0.022] |  |  | [-0.035,0.004] |
|  |  |  |  |  |  |
| Year FE | Yes | Yes | Yes | Yes | Yes |
| *N* | 78200 | 69158 | 53570 | 45862 | 44064 |
| Differential Trend | No | Yes | No | No | Yes |
| CEM Weights | No | No | Yes | Yes | Yes |

95% confidence intervals in brackets

Table F Robustness checks (Coarsened Exact Matching and DDD Estimates) for FoodSwitch data on the outcome:Saturated Fat (g/100g or ml)

|  | (1) | (2) | (3) | (4) | (5) |
| --- | --- | --- | --- | --- | --- |
|  | Baseline | Diff. Trend | CEM Weights: Nutrient Info Only | CEM Weights: Nutrient Info + Food Group | CEM Weights with Food Group + Trend |
| HSR Reformulation Effect | 0.030 | 0.029 | 0.001 | 0.028 | 0.034 |
|  | [-0.013,0.074] | [-0.016,0.074] | [-0.040,0.042] | [-0.019,0.076] | [-0.008,0.076] |
|  |  |  |  |  |  |
| Diff. trend. |  | 0.001 |  |  | -0.002 |
|  |  | [-0.015,0.016] |  |  | [-0.020,0.017] |
|  |  |  |  |  |  |
| Year FE | Yes | Yes | Yes | Yes | Yes |
| *N* | 77933 | 68892 | 53437 | 45754 | 43957 |
| Differential Trend | No | Yes | No | No | Yes |
| CEM Weights | No | No | Yes | Yes | Yes |

95% confidence intervals in brackets

Table G Robustness checks (Coarsened Exact Matching and DDD Estimates) for FoodSwitch data on the outcome: Fibre (g/100g or ml)

|  | (1) | (2) | (3) | (4) | (5) |
| --- | --- | --- | --- | --- | --- |
|  | Baseline | Diff. Trend | CEM Weights: Nutrient Info Only | CEM Weights: Nutrient Info + Food Group | CEM Weights with Food Group + Trend |
| HSR Reformulation Effect | -0.041 | -0.023 | 0.015 | 0.020 | 0.003 |
|  | [-0.081,-0.000] | [-0.071,0.025] | [-0.017,0.046] | [-0.011,0.051] | [-0.039,0.044] |
|  |  |  |  |  |  |
| Diff. trend. |  | -0.007 |  |  | 0.009 |
|  |  | [-0.021,0.006] |  |  | [-0.003,0.021] |
|  |  |  |  |  |  |
| Year FE | Yes | Yes | Yes | Yes | Yes |
| *N* | 78339 | 69296 | 53582 | 45871 | 44073 |
| Differential Trend | No | Yes | No | No | Yes |
| CEM Weights | No | No | Yes | Yes | Yes |

95% confidence intervals in brackets

### New Zealand

Table H Robustness checks (Coarsened Exact Matching and DDD Estimates) for Nutritrack data on the outcome: Stars

|  | (1) | (2) | (3) | (4) | (5) |
| --- | --- | --- | --- | --- | --- |
|  | Baseline | Diff. Trend | CEM Weights: Nutrient Info Only | CEM Weights: Nutrient Info + Food Group | CEM Weights with Food Group + Trend |
| HSR Reformulation Effect | 0.067 | 0.069 | 0.077 | 0.075 | 0.066 |
|  | [0.051,0.082] | [0.053,0.085] | [0.062,0.092] | [0.059,0.090] | [0.050,0.082] |
|  |  |  |  |  |  |
| Diff. Trend |  | -0.000 |  |  | 0.004 |
|  |  | [-0.004,0.004] |  |  | [-0.000,0.007] |
|  |  |  |  |  |  |
| Year FE | Yes | Yes | Yes | Yes | Yes |
| *N* | 90089 | 85693 | 86180 | 83686 | 83138 |
| Differential Trend | No | Yes | No | No | Yes |
| CEM Weights | No | No | Yes | Yes | Yes |

95% confidence intervals in brackets

Table I Robustness checks (Coarsened Exact Matching and DDD Estimates) for Nutritrack data on the outcome:Energy (kJ/100g or ml)

|  | (1) | (2) | (3) | (4) | (5) |
| --- | --- | --- | --- | --- | --- |
|  | Baseline | Diff. Trend | CEM Weights: Nutrient Info Only | CEM Weights: Nutrient Info + Food Group | CEM Weights with Food Group + Trend |
| HSR Reformulation Effect | -0.691 | -1.949 | -2.429 | -0.325 | -1.509 |
|  | [-5.145,3.763] | [-6.656,2.759] | [-7.046,2.187] | [-4.864,4.215] | [-6.220,3.201] |
|  |  |  |  |  |  |
| Diff. Trend |  | 0.636 |  |  | 0.531 |
|  |  | [-0.765,2.037] |  |  | [-0.873,1.935] |
|  |  |  |  |  |  |
| Year FE | Yes | Yes | Yes | Yes | Yes |
| *N* | 93377 | 88936 | 89421 | 86265 | 85715 |
| Differential Trend | No | Yes | No | No | Yes |
| CEM Weights | No | No | Yes | Yes | Yes |

95% confidence intervals in brackets

Table J Robustness checks (Coarsened Exact Matching and DDD Estimates) for Nutritrack data on the outcome:Sodium (mg/100g or ml)

|  | (1) | (2) | (3) | (4) | (5) |
| --- | --- | --- | --- | --- | --- |
|  | Baseline | Diff. Trend | CEM Weights: Nutrient Info Only | CEM Weights: Nutrient Info + Food Group | CEM Weights with Food Group + Trend |
| HSR Reformulation Effect | -16.165 | -18.603 | -21.528 | -18.856 | -16.558 |
|  | [-25.392,-6.938] | [-27.335,-9.872] | [-31.680,-11.376] | [-28.380,-9.332] | [-25.023,-8.092] |
|  |  |  |  |  |  |
| Diff. Trend |  | 1.349 |  |  | -0.512 |
|  |  | [-2.844,5.542] |  |  | [-4.558,3.533] |
|  |  |  |  |  |  |
| Year FE | Yes | Yes | Yes | Yes | Yes |
| *N* | 92889 | 88439 |  | 85896 | 85344 |
| Differential Trend | No | Yes |  | No | Yes |
| CEM Weights | No | No |  | Yes | Yes |

95% confidence intervals in brackets

Table K Robustness checks (Coarsened Exact Matching and DDD Estimates) for Nutritrack data on the outcome:Sugar (g/100g or ml)

|  | (1) | (2) | (3) | (4) | (5) |
| --- | --- | --- | --- | --- | --- |
|  | Baseline | Diff. Trend | CEM Weights: Nutrient Info Only | CEM Weights: Nutrient Info + Food Group | CEM Weights with Food Group + Trend |
| HSR Reformulation Effect | -0.211 | -0.199 | -0.290 | -0.221 | -0.176 |
|  | [-0.335,-0.087] | [-0.329,-0.070] | [-0.415,-0.166] | [-0.344,-0.099] | [-0.305,-0.048] |
|  |  |  |  |  |  |
| Diff. Trend |  | -0.010 |  |  | -0.023 |
|  |  | [-0.039,0.018] |  |  | [-0.050,0.005] |
|  |  |  |  |  |  |
| Year FE | Yes | Yes | Yes | Yes | Yes |
| *N* | 92633 | 88217 | 88705 | 85708 | 85156 |
| Differential Trend | No | Yes | No | No | Yes |
| CEM Weights | No | No | Yes | Yes | Yes |

95% confidence intervals in brackets

Table L Robustness checks (Coarsened Exact Matching and DDD Estimates) for Nutritrack data on the outcome: Protein (g/100g or ml)

|  | (1) | (2) | (3) | (4) | (5) |
| --- | --- | --- | --- | --- | --- |
|  | Baseline | Diff. Trend | CEM Weights: Nutrient Info Only | CEM Weights: Nutrient Info + Food Group | CEM Weights with Food Group + Trend |
| HSR Reformulation Effect | -0.021 | 0.006 | -0.037 | -0.022 | 0.006 |
|  | [-0.078,0.035] | [-0.057,0.069] | [-0.097,0.023] | [-0.081,0.036] | [-0.058,0.070] |
|  |  |  |  |  |  |
| Diff. Trend |  | -0.007 |  |  | -0.008 |
|  |  | [-0.022,0.008] |  |  | [-0.024,0.008] |
|  |  |  |  |  |  |
| Year FE | Yes | Yes | Yes | Yes | Yes |
| *N* | 93372 | 88929 | 89416 | 86255 | 85703 |
| Differential Trend | No | Yes | No | No | Yes |
| CEM Weights | No | No | Yes | Yes | Yes |

95% confidence intervals in brackets

Table M Robustness checks (Coarsened Exact Matching and DDD Estimates) for Nutritrack data on the outcome:Saturated Fat (g/100g or ml)

|  | (1) | (2) | (3) | (4) | (5) |
| --- | --- | --- | --- | --- | --- |
|  | Baseline | Diff. Trend | CEM Weights: Nutrient Info Only | CEM Weights: Nutrient Info + Food Group | CEM Weights with Food Group + Trend |
| HSR Reformulation Effect | -0.040 | -0.041 | -0.061 | -0.056 | -0.039 |
|  | [-0.093,0.014] | [-0.095,0.013] | [-0.114,-0.009] | [-0.108,-0.003] | [-0.093,0.016] |
|  |  |  |  |  |  |
| Diff. Trend |  | -0.000 |  |  | -0.006 |
|  |  | [-0.009,0.009] |  |  | [-0.015,0.003] |
|  |  |  |  |  |  |
| Year FE | Yes | Yes | Yes | Yes | Yes |
| *N* | 93348 | 88904 | 89392 | 86234 | 85681 |
| Differential Trend | No | Yes | No | No | Yes |
| CEM Weights | No | No | Yes | Yes | Yes |

95% confidence intervals in brackets

Table N Robustness checks (Coarsened Exact Matching and DDD Estimates) for Nutritrack data on the outcome:Fibre (g/100g or ml)

|  | (1) | (2) | (3) | (4) | (5) |
| --- | --- | --- | --- | --- | --- |
|  | Baseline | Diff. Trend | CEM Weights: Nutrient Info Only | CEM Weights: Nutrient Info + Food Group | CEM Weights with Food Group + Trend |
| HSR Reformulation Effect | 0.041 | 0.042 | 0.033 | 0.037 | 0.038 |
|  | [0.005,0.077] | [0.006,0.077] | [-0.006,0.073] | [-0.003,0.076] | [0.002,0.074] |
|  |  |  |  |  |  |
| Diff. Trend |  | 0.004 |  |  | 0.003 |
|  |  | [-0.006,0.013] |  |  | [-0.008,0.013] |
|  |  |  |  |  |  |
| Year FE | Yes | Yes | Yes | Yes | Yes |
| *N* | 95239 | 90741 | 91235 | 87843 | 87284 |
| Differential Trend | No | Yes | No | No | Yes |
| CEM Weights | No | No | Yes | Yes | Yes |

95% confidence intervals in brackets
