# Supplementary material for: The impact of voluntary front-of-pack nutrition labelling on packaged food reformulation: A difference-in-differences analysis of the Australasian Health Star Rating scheme
Source: PLoS Med. 2020 Nov 20;17(11):e1003427. doi: 10.1371/journal.pmed.1003427 (PMC7679009; doi:10.1371/journal.pmed.1003427)
Supplement: S6 Text — (DOCX) [file pmed.1003427.s006.docx]

# The Impact of Voluntary Front of Pack Nutrition Labelling on Packaged Food Reformulation

## S6 Text: Effects of fibre imputation

### Australia

Table A: Effect of fibre imputations on Australian data

|  | (1) | (3) |
| --- | --- | --- |
|  | Without imputation | Final Imputations |
| Fibre reformulation effect | -0.035 | -0.039 |
|  | [-0.134,0.063] | [-0.080,0.000] |
| *N* | 27955 | 78339 |
| 95% confidence intervals in brackets |  |  |

### New Zealand

Table B: Effects of fibre imputation on New Zealand data

|  | (1) | (2) | (3) |
| --- | --- | --- | --- |
|  | Nutritrack with False Zeros | False zeros removed | Final Imputations |
| Fibre reformulation effect | 0.414 | 0.095 | 0.041 |
|  | [0.257,0.571] | [-0.025,0.215] | [0.004,0.077] |
| *N* | 38381 | 25535 | 95239 |
| 95% confidence intervals in brackets | | | |

### 
