# Supplementary material for: The impact of voluntary front-of-pack nutrition labelling on packaged food reformulation: A difference-in-differences analysis of the Australasian Health Star Rating scheme
Source: PLoS Med. 2020 Nov 20;17(11):e1003427. doi: 10.1371/journal.pmed.1003427 (PMC7679009; doi:10.1371/journal.pmed.1003427)
Supplement: S7 Text — (DOCX) [file pmed.1003427.s007.docx]

## S7 Text: T-test between most nutritious (4.0-5.0) and remaining categories (0.5-1.5), (2.0-3.5)

### Australia

Table A: T-test between most nutritious (4.0-5.0) and remaining categories (0.5-1.5), (2.0-3.5) for Australia

|  | (1) | (2) | (3) | (4) | (5) | (6) | (7) |
| --- | --- | --- | --- | --- | --- | --- | --- |
|  | HSR rating | Energy | Sodium | Sugar | Protein | Sat. Fat | Fibre |
| 4.0-5.0 | -0.035 | 0.951 | 1.620 | 0.082 | -0.026 | 0.107 | -0.089 |
|  | [-0.056,-0.014] | [-3.358,5.261] | [-3.287,6.527] | [-0.017,0.181] | [-0.118,0.065] | [0.066,0.147] | [-0.196,0.019] |
| **Standardised Difference between most nutritious (4.0-5.0) and:** | | | | | | | |
| 0.5-1.5 | 0.161 | -14.742 | -13.726 | -0.804 | -0.053 | -0.311 | 0.173 |
|  | [0.123,0.198] | [-24.486,-4.999] | [-29.367,1.914] | [-1.136,-0.472] | [-0.184,0.079] | [-0.462,-0.160] | [0.046,0.301] |
| 2.0-3.5 | 0.070 | -5.457 | -6.799 | -0.077 | 0.056 | -0.035 | 0.039 |
|  | [0.042,0.097] | [-11.280,0.365] | [-11.583,-2.016] | [-0.251,0.097] | [-0.046,0.158] | [-0.099,0.029] | [-0.076,0.154] |
| Year Dummies | Yes | Yes | Yes | Yes | Yes | Yes | Yes |
| *N* | 70680 | 70796 | 70849 | 70787 | 70781 | 70740 | 70849 |

95% confidence intervals in brackets

### New Zealand

Table B: T-test between most nutritious (4.0-5.0) and remaining categories (0.5-1.5), (2.0-3.5) for New Zealand

|  | (1) | (2) | (3) | (4) | (5) | (6) | (7) |
| --- | --- | --- | --- | --- | --- | --- | --- |
|  | HSR rating | Energy | Sodium | Sugar | Protein | Sat. Fat | Fibre |
| 4.0-5.0 | 0.014 | 3.927 | -8.032 | -0.071 | -0.021 | 0.030 | 0.029 |
|  | [-0.002,0.029] | [-2.004,9.858] | [-13.922,-2.142] | [-0.201,0.060] | [-0.116,0.073] | [-0.010,0.070] | [-0.041,0.099] |
| **Standardised Difference between most nutritious (4.0-5.0) and:** | | | | | | | |
| 0.5-1.5 | 0.087 | -6.127 | -44.892 | -0.387 | 0.016 | -0.118 | 0.002 |
|  | [0.048,0.127] | [-18.294,6.041] | [-82.656,-7.127] | [-0.901,0.126] | [-0.154,0.186] | [-0.427,0.191] | [-0.076,0.079] |
| 2.0-3.5 | 0.098 | -8.562 | -6.342 | -0.211 | -0.008 | -0.125 | 0.035 |
|  | [0.066,0.130] | [-17.095,-0.030] | [-22.824,10.139] | [-0.414,-0.008] | [-0.121,0.105] | [-0.197,-0.053] | [-0.045,0.114] |
| Year Dummies | Yes | Yes | Yes | Yes | Yes | Yes | Yes |
| *N* | 86210 | 87431 | 87027 | 87021 | 87431 | 87402 | 88232 |

95% confidence intervals in brackets
