# Supplementary material for: The impact of voluntary front-of-pack nutrition labelling on packaged food reformulation: A difference-in-differences analysis of the Australasian Health Star Rating scheme
Source: PLoS Med. 2020 Nov 20;17(11):e1003427. doi: 10.1371/journal.pmed.1003427 (PMC7679009; doi:10.1371/journal.pmed.1003427)
Supplement: S8 Text — (DOCX) [file pmed.1003427.s008.docx]

**The Impact of Voluntary Front of Pack Nutrition Labelling on Packaged Food Reformulation**

**S8 Text: STROBE Reporting Matrix**

The Impact of Voluntary Front of Pack Nutrition Labelling on Packaged Food Reformulation: A difference-in-differences analysis of the Australasian Health Star Rating scheme

STROBE Statement—checklist of items that should be included in reports of observational studies

|  | Item No | Recommendation and Matching Excerpt | Reference in paper |
| --- | --- | --- | --- |
| **Title and abstract** | 1 | (*a*) Indicate the study’s design with a commonly used term in the title or the abstract  “**A difference-in-differences** analysis of the Australasian Health Star Rating scheme” | Title |
|  |  | (*b*) Provide in the abstract an informative and balanced summary of what was done and what was found Methods and Findings Annual nutrition information panel data was collected for non-seasonal packaged foods sold in major supermarkets in Auckland from 2013-19, and Sydney from 2014-18… Initially unhealthy products showed larger reformulation effects when adopting HSR than healthier products. | Abstract: Methods and Findings |
| Introduction | | |  |
| Background/rationale | 2 | Explain the scientific background and rationale for the investigation being reported  There is limited evidence on the effectiveness of FoPL policies on food reformulation, and what evidence exists traverses a variety of FoPL systems… Generally, the relatively low adoption rates of HSR in 2016 (between 5 to 10 per-cent of all packaged foods) may impact the reliability of these estimates. | Introduction: Para. 2 and 3 |
| Objectives | 3 | State specific objectives, including any prespecified hypotheses  This study, therefore, evaluated the effect of HSR on packaged food reformulation using panel data techniques (10). It also analysed differences in reformulation by baseline product healthiness. | Introduction: Para 4 |
| Methods | | |  |
| Study design | 4 | Present key elements of study design early in the paper Study overview The primary data sources on food formulation were mandatory back-of-pack Nutrition Information panel (NIP) data from both Australia (2014-2018) and NZ (2013-2019) on non-seasonal packaged products sold at major supermarkets in both countries. Using a difference-in-differences design with product- and time-level fixed effects, we controlled for both within-product confounding and market-wide reformulation effects that may have biased the results of our study. The outcomes examined were changes in: the density of the four targeted negative nutrients in HSR – energy, sugars, saturated fat, & sodium; and two positive constituents – protein, and fibre. Another outcome was the HSR Rating score itself, which we imputed for all products at all time points using NIP data and the publicly available HSR algorithm. Last, we examined heterogeneous effects of HSR adoption by the healthiness of products defined using their imputed HSR ratings, *before* HSR label adoption. | Methods – Study Overview |
| Setting | 5 | Describe the setting, locations, and relevant dates, including periods of recruitment, exposure, follow-up, and data collection  Data on the nutritional composition of non-seasonal packaged food products (Stock Keeping Units [SKUs]) sold across several Australasian supermarkets was taken from two sources: Nutritrack 2013-19 data for NZ, collected by the National Institute of Health Innovation at the University of Auckland (11); and FoodSwitch 2014-18 data for Australia, collected by The George Institute for Global Health (12) | Methods: Data Sources – Paragraph 1 |
| Participants | 6 | (*a*) *Cohort study*—Give the eligibility criteria, and the sources and methods of selection of participants. Describe methods of follow-up  *Case-control study*—Give the eligibility criteria, and the sources and methods of case ascertainment and control selection. Give the rationale for the choice of cases and controls  *Cross-sectional study*—Give the eligibility criteria, and the sources and methods of selection of participants  Nutritrack collects information between February and April each year on all packaged food and beverages sold at four major supermarkets in Auckland, NZ; one store each of New World, 4Square, Countdown, and PAK’nSAVE chains. Key exclusions from the dataset include products that do not display a nutrient information panel (NIP), unpackaged fresh foods, bulk bin items, alcohol, seasonal products (such as Easter eggs), and dietary supplements.  Similarly, FoodSwitch collects annual data of product information from four stores in Sydney, Australia; one each of ALDI, Coles, IGA, and Woolworths chains. This field survey data is augmented by supplementary data collection and crowdsourcing through the FoodSwitch mobile app which has been downloaded over 600,000 times. | Methods: Data Sources – Paragraph 2,3,4,5 |
|  |  | (*b*) *Cohort study*—For matched studies, give matching criteria and number of exposed and unexposed  *Case-control study*—For matched studies, give matching criteria and the number of controls per case |  |
| Variables | 7 | Clearly define all outcomes, exposures, predictors, potential confounders, and effect modifiers. Give diagnostic criteria, if applicable Exposure and Outcome Variables Both data sets contained SKU-specific barcodes, which were used to link product information over time, forming two longitudinal country-specific datasets.. To enable a consistent before-after analysis, all analyses on HSR ratings used imputed HSR scores, and the 'actual' displayed HSR was not used. | Methods: Data Sources: Exposure and Outcome Variables |
| Data sources/ measurement | 8* | For each variable of interest, give sources of data and details of methods of assessment (measurement). Describe comparability of assessment methods if there is more than one group Data Sources Data on the nutritional composition of non-seasonal packaged food products (Stock Keeping Units [SKUs]) sold across several Australasian supermarkets … This field survey data is augmented by supplementary data collection and crowdsourcing through the FoodSwitch mobile app which has been downloaded over 600,000 times. | *Methods: Data Sources* |
| Bias | 9 | Describe any efforts to address potential sources of bias  We used a difference-in-differences design, based on a before-after difference in levels for the outcome variable, in the presence of a comparison group to control for factors that would have affected the treatment group in the absence of treatment…. **Sensitivity Analyses** We conducted several robustness checks… | Methods: Analysis – Para 1;  Methods: Analysis -Sensitivity Analyses |
| Study size | 10 | Explain how the study size was arrived at | NA |
| Quantitative variables | 11 | Explain how quantitative variables were handled in the analyses. If applicable, describe which groupings were chosen and why **Exposure and Outcome Variables** Both data sets contained SKU-specific barcodes, which were used to link product information over time, forming two longitudinal country-specific datasets. To enable a consistent before-after analysis, all analyses on HSR ratings used imputed HSR scores, and the 'actual' displayed HSR was not used. | Methods: Data Sources: Exposure and Outcome Variables |
| Statistical methods | 12 | 1. Describe all statistical methods, including those used to control for confounding  Analysis We used a difference-in-differences design, based on a before-after difference in levels for the outcome variable, in the presence of a comparison group to control for factors that would have affected the treatment group in the absence of treatment… | Methods: Analysis – Para 1;  Methods: Analysis -Sensitivity Analyses |
|  |  | (*b*) Describe any methods used to examine subgroups and interactions Stratification by pre-labelling imputed HSR rating Products were split into three categories based on their baseline imputed HSR rating (before any adopted HSR labelling): 0.5 to 1.5, 2.0 to 3.5, and 4.0 to 5.0 stars. Category indicators were interacted with HSR participation in equation (1) to estimate differential reformulation effects by pre-intervention HSR. | Methods: Analysis: Stratification by pre-labelling imputed HSR rating |
|  |  | (*c*) Explain how missing data were addressed  However, fibre was not mandatory on product NIPs and was displayed on roughly 40% of all observations in the sample frame. In some cases, fibre values were entered as zero when they were missing. In such instances, these zeros were changed to missing. To maximise overall sample sizes, missing fibre values were imputed with leads and lags, i.e. if there was no evidence of product reformulation on other nutrients, then data on a missing fibre in one year was imputed with the value from an adjacent year. | Methods: Exposure and Outcome Variables: Para 2 |
|  |  | (*d*) *Cohort study*—If applicable, explain how loss to follow-up was addressed  *Case-control study*—If applicable, explain how matching of cases and controls was addressed  *Cross-sectional study*—If applicable, describe analytical methods taking account of sampling strategy  Case Control: We used a difference-in-differences design, based on a before-after difference in levels for the outcome variable, in the presence of a comparison group to control for factors that would have affected the treatment group in the absence of treatment. … First, coarsened exact matching (CEM) is a non-parametric matching technique that balances pre-labelling nutrient information between HSR products and products that never received HSR labelling (15). CEM generated matching weights were used in equation (1): | Methods: Analysis – Para 1;  Methods: Analysis -Sensitivity Analyses: Para 1 |
|  |  | (*e*) Describe any sensitivity analyses Sensitivity Analyses We conducted several robustness checks… Last, we combined CEM and differential trend approaches. | Methods: Analysis -Sensitivity Analyses: |

| Results | | | Page# | |
| --- | --- | --- | --- | --- |
| Participants | 13* | 1. Report numbers of individuals at each stage of study—eg numbers potentially eligible, examined for eligibility, confirmed eligible, included in the study, completing follow-up, and analysed   Ns reported on each table | Table 1, 2, 3 |  |
|  |  | 1. Give reasons for non-participation at each stage   Key exclusions from the dataset include products that do not display a nutrient information panel (NIP), unpackaged fresh foods, bulk bin items, alcohol, seasonal products (such as Easter eggs), and dietary supplements. | Methods: Data Sources: Para 2,5 |  |
|  |  | (c) Consider use of a flow diagram | NA |  |
| Descriptive data | 14* | 1. Give characteristics of study participants (eg demographic, clinical, social) and information on exposures and potential confounders   Table 1 gives the nutritional composition at baseline for products that did not adopt HSR (Column 1), and products that adopted HSR labelling, in the year before labelling (Column 2, treated group). | Table 1 |  |
|  |  | (b) Indicate number of participants with missing data for each variable of interest | NA |  |
|  |  | (c) *Cohort study*—Summarise follow-up time (eg, average and total amount) | NA |  |
| Outcome data | 15* | *Cohort study*—Report numbers of outcome events or summary measures over time |  |  |
|  |  | *Case-control study—*Report numbers in each exposure category, or summary measures of exposure  *Ns reported in each table* | *Table 1, 2, 3; Supplement S1* |  |
|  |  | *Cross-sectional study—*Report numbers of outcome events or summary measures |  |  |
| Main results | 16 | 1. Give unadjusted estimates and, if applicable, confounder-adjusted estimates and their precision (eg, 95% confidence interval). Make clear which confounders were adjusted for and why they were included   Table 2: Effect of voluntary adoption of HSR label on nutrient profile score and component nutrients, controlling for market-wide trends and time-invariant product level characteristics using fixed effects analysis. (Note: 95% CI in brackets; + p < 0.10, * p < 0.05, ** p < 0.01, *** p < 0.001) | Table 2 |  |
|  |  | (*b*) Report category boundaries when continuous variables were categorized | NA |  |
|  |  | (*c*) If relevant, consider translating estimates of relative risk into absolute risk for a meaningful time period | NA |  |
| Other analyses | 17 | Report other analyses done—eg analyses of subgroups and interactions, and sensitivity analyses Sensitivity Analyses Figures of pre-existing trends in reformulation between labelled and unlabelled products are presented in Appendix S2. Generally, we did not find differences in reformulation trends before labelling, strengthening the validity of the parallel paths identifying assumption for differences-in-differences.  Effect sizes were generally marginally larger when we use CEM matching weights in equation 1, and marginally lower when allowing for differential pre-treatment reformulation trends (Appendix S3). They were also similar when we combined the two approaches. These analyses supported the validity of the difference-in-difference estimates presented above. | Results: Stratification by pre-treatment imputed HSR rating  Results: Sensitivity Analyses |  |
| Discussion | | |  |  |
| Key results | 18 | Summarise key results with reference to study objectives  The voluntary adoption of the HSR moderately affected product reformulation… This was consistent with ceiling effects in reformulation, whereby already nutritious products have limited scope for healthier reformulation. | Discussion: Para 1 |  |
| Limitations | 19 | Discuss limitations of the study, taking into account sources of potential bias or imprecision. Discuss both direction and magnitude of any potential bias  Limitations arise from our imputations of missing values…Results were robust to these checks (S3 Appendix). | Discussion: Strengths and Limitations  Para 2, 3,4 |  |
| Interpretation | 20 | Give a cautious overall interpretation of results considering objectives, limitations, multiplicity of analyses, results from similar studies, and other relevant evidence Implications HSR, a voluntary FOPL scheme, drives small industry-led reformulation. … To enhance the evidence of HSR or comparable graded summary FoPL on affecting consumer behaviour, we are conducting a follow-on study analysing the effect of HSR on purchasing patterns using household panel data. | Discussion: Implications |  |
| Generalisability | 21 | Discuss the generalisability (external validity) of the study results  FoPL systems include a wide range of designs and policy, cautioning against simple comparisons of the results of different studies. However, our results serve as a relevant benchmark for similar graded summary schemes, such as Nutri-score (17), which has seen increasing uptake across Europe. | Discussion: Comparison with Other Studies |  |
| Other information | | |  |  |
| Funding | 22 | Give the source of funding and the role of the funders for the present study and, if applicable, for the original study on which the present article is based  This was an investigator-initiated study funded by a Health Research Council of New Zealand programme grant (18/672). The Health Research Council (http://www.hrc.govt.nz/) had no role in design, analysis, interpretation or reporting of these analyses. | Abstract: Funding |  |

*Give information separately for cases and controls in case-control studies and, if applicable, for exposed and unexposed groups in cohort and cross-sectional studies.

**Note:** An Explanation and Elaboration article discusses each checklist item and gives methodological background and published examples of transparent reporting. The STROBE checklist is best used in conjunction with this article (freely available on the Web sites of PLoS Medicine at http://www.plosmedicine.org/, Annals of Internal Medicine at http://www.annals.org/, and Epidemiology at http://www.epidem.com/). Information on the STROBE Initiative is available at www.strobe-statement.org.
