# Supplementary material for: The impact of voluntary front-of-pack nutrition labelling on packaged food reformulation: A difference-in-differences analysis of the Australasian Health Star Rating scheme
Source: PLoS Med. 2020 Nov 20;17(11):e1003427. doi: 10.1371/journal.pmed.1003427 (PMC7679009; doi:10.1371/journal.pmed.1003427)
Supplement: S9 Text — (DOCX) [file pmed.1003427.s009.docx]

The Impact of Voluntary Front of Pack Nutrition Labelling on Packaged Food Reformulation

## S9 Text: Grant Extract

The following text was taken from the HRCNZ Grant 18-672, which funded the study. Portions of this grant not corresponding to this article - those that describe commercial data, researcher credentials, or research outside the scope of this study, have been removed. The health star logo in Fig A could not be included due to copyright license conflicts with PLOS Medicine’s open access CC-BY license.

**OBJECTIVE 3 - Measure the impact of front-of-pack nutrition labels on population diets**

The aim is to evaluate the impact of Health Star Rating (HSR) nutrition labels over eight years (2013-

2020) on: the nutritional composition and reformulation of packaged foods (Research Question (RQ) 1)

**Rationale**

In 2014, New Zealand and Australia adopted a new voluntary, interpretive front-of-pack nutrition labelling system, the HSR. HSR rates the nutrition content of packaged food in half-star increments from half a star (least healthy) to five stars (most healthy) (Fig A). The number of stars displayed is calculated based on the energy, saturated fat, total sugar, sodium, and fruit, vegetable, nut and legume (FVNL) levels and, in some instances, protein and fibre content^4^. Labelling systems like HSR guide healthier food choices^5,6^, especially amongst more nutrition-conscious shoppers^1^.

[FIGURE OMITTED DUE TO COPYRIGHT ISSUES]

**Fig A: Health Star Rating label**

Importantly, evidence suggests that interpretive labels may

also improve population diets through healthier product reformulation by the food industry. Adoption of the Choices nutrition logo in the Netherlands^7^, the Health Check Program symbol in Canada^8^, and the Pick the Tick logo in New Zealand^9^ and Australia^10^ all led to reported reformulation of selected food products on the market. The adoption of HSR in 2014 by NZ and Australia has created a natural experiment that could have important effects on population diets. The research we propose is worldleading because we have comprehensive data to estimate the impact of labelling on reformulation of all packaged foods (not just a select sample) *and* household food purchasing behaviour, thus enabling us to estimate effects on total population diet. **Pilot data**

In 2016, we undertook a simple, interim (two-year) analysis of HSR^2^. Annual surveys of packaged food and beverage labelling and composition were undertaken in supermarkets before and after adoption of HSR i.e., 2014 to 2016. Small but statistically significant changes were observed in mean energy density (−29 KJ/100 g, 95%CI -48 to -11), sodium (−49 mg/100 g, 95%CI -5 to -96; ) and fibre (+0.5 g/100 g, 95%CI 0.2 to 0.8) contents of HSR-labelled products compared with their composition prior to adoption of HSR2.


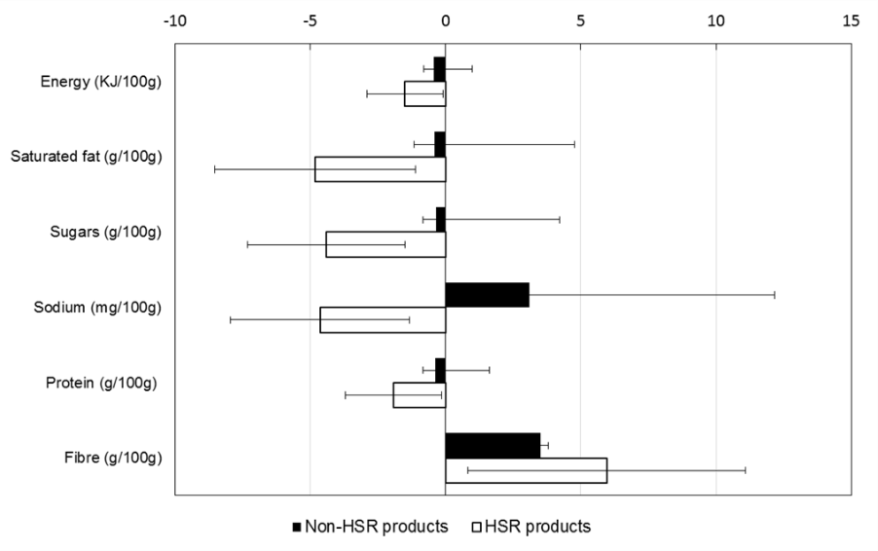
Reformulation of HSR-labelled products was greater than that of non-HSR-labelled products over the same period, e.g., sodium content of HSR products decreased by 4.6% while that of non-HSR products increased by 3.1% (Fig B). This short-term, simple before and after analysis suggests that rollout of the HSR is driving healthier reformulation of some products. However, we were unable to determine longterm reformulation effects, changes in formulation of foods other than early adopters, or

effects on consumer food **Fig B: % change in nutrient composition between 2014** purchasing behaviour (e.g. **and 2016 for HSR and non-HSR products** compensatory purchasing).

**Design**

It is not always possible to undertake ‘gold-standard’ RCTs for population-level interventions, and RCTs sometimes lack external validity^11^. Natural experiment methods are often recommended in this situation, and there is a long tradition of the approach in epidemiology and econometrics^12^. Natural experiment approaches evaluate the introduction of a policy that has a (approximate) random element (i.e. with little or no systematic correlation with confounding factors like price changes, seasonality, other policy changes). In the case of HSR, the system was instituted nationally, but with variation over time when products started displaying HSR labels since uptake by industry is voluntary. Such a natural experiment can provide convincing evidence of impact even when effects are small or take time to appear if the scale of intervention is large and data available rich.^13^ A range of modelling methods and analyses are available, such as difference in difference (DiD) and fixed effects models. These methods have been used previously in other countries to evaluate the effects of taxes on sugar-sweetened beverage consumption^14^, healthy food initiatives conducted by supermarket retailers^15^, and voluntary pledges by the food industry to reduce the energy density of the food supply^16^. We have data on all packaged foods available in NZ supermarkets in each year in Nutritrack. We propose to use fixed effects modelling methods, as they adjust for all time invariant confounding (observed and unobserved), allow ‘open’ cohorts of shoppers and products entering and leaving the panel, allow adjustment for measured time-varying confounders (e.g. seasonality, income), and can be specified in the range of ways possible with a regression model^17-19^.

**Methods**

*RQ1 How much does the nutrient profile of packaged foods change due to industry reformulation following uptake of HSR?* Nutritrack data for both NZ and Australia will be used (see Platform section above). Overall nutritional composition of foods will be measured using nutrient profile scores^3^ for each food, which are calculated based on energy, saturated fat, total sugar, and sodium content per 100g; as well as the amount of fruits, vegetables, nuts and legumes, protein and fibre. There are multiple ways to group foods, opening a risk to data-mining. Accordingly, we will a priori define groups of foods based on dietary similarities and HSR status (in particular whether in time the HSR was universally applied to the group) before undertaking final analyses. Analyses will be restricted to: (1) Food products that began displaying a HSR label sometime during the study period; and (2) Food products on the market at least one year preceding, and at least one-year following, the introduction of the HSR on *that* product. We will then describe (using e.g. line plots, tables) a range of nutrient profile measures by year of HSR introduction and by food category, looking for a change in nutrient profile from pre- to post HSR, followed by fixed effects regression modelling to quantify (any) impact. Our pilot data demonstrates ample power for estimation of an overall effect given that there was only 5.3% HSR label uptake in 2016^2^ (note HSR uptake was 14% in our 2017 Nutritrack data collection, and industry and government sources suggest the upward trend will continue. We will also test multiple subsidiary research questions:

- Does any effect of HSR on reformulation vary over time? (i.e. early adopter products may be more easily reformulated) - *Include interaction terms for year in FE modelling*
- Does any effect of HSR on reformulation vary by baseline nutrient profile? (i.e. are improvements in composition (if any) seen across all products, or only those (un)healthy to begin with? - *Include interaction terms of pre-post dummy with baseline nutrient profile*
- Does the nutrient profile of products displaying a HSR label continue to improve over time relative to non-labelled products, or do unlabelled products catch up? (i.e. spill over effects) - *Include calendar year interactions (‘slopes’) over multiple years with HSR-dummy.*

REFERENCES

| 1. | Ni Mhurchu C, Volkova E, Jiang Y, Eyles H, Michie J, Neal B, Blakely T, Swinburn B, Rayner M. Effects of interpretive nutrition labels on consumer food purchases: the Starlight randomized controlled trial. *American Journal of Clinical Nutrition.* 2017;105:695-704. |
| --- | --- |
| 2. | Ni Mhurchu C, Eyles H, Choi Y. Effects of a voluntary front-of-pack nutrition labelling system on packaged food reformulation: The Health Star Rating system in New Zealand. *Nutrients.* 2017;9:918 doi:910.3390/nu9080918. |

3. Food Standards Australia New Zealand. *Short guide for industry to the Nutrient Profiling Scoring Criterion (NPSC) in Standard 1.2.7 - Nutrition, health and related claims.* Wellington: Food Standards Australia New Zealand;2013.

1. Food Standards Australia New Zealand. *Guide for industry to the Health Star Rating Calculator (HSRC).* Canberra: Food Standards Australia New Zealand;2015.
2. Rahkovsky I, Lin B-H, Lin C, Lee Y. Effects of the Guiding Stars Program on purchases of ready-to-eat cereals with different nutritional attributes. *Food Policy.* 2013;43:100-107.
3. Sutherland LA, Kaley LA, Fischer L. Guiding Stars: the effect of a nutrition navigation program on consumer purchases at the supermarket. *American Journal of Clinical Nutrition.* April 1, 2010 2010;91(4):1090S-1094S.
4. Vyth E, Steenhuis I, Roodenburg A, Brug J, Seidell J. Front-of-pack nutrition label stimulates healthier product development: a quantitative analysis. *International Journal of Behavioral Nutrition & Physical Activity.* 2010;7 65.
5. Dummer J. Sodium reduction in Canadian food products with the Health Check Program. *Canadian Journal of Dietetic Practice and Research.* 2012;73:e227-e232.

9. Young L, Swinburn B. Impact of the Pick the Tick food information programme on the salt content of food in New Zealand. *Health Promotion International.* Mar 2002;17(1):13-19.

1. Williams P, McMahon A, Bousted R. A case study of sodium reduction in breakfast cereals and the impact of the Pick the Tick food information program in Australia. Health Promotion International. 2003;18(1):51-56.
2. Frieden TR. Evidence for Health Decision Making — Beyond Randomized, Controlled Trials. New England Journal of Medicine. 2017;377(5):465-475.
3. Davey-Smith G. Behind the Broad Street pump: aetiology, epidemiology and prevention of cholera in mid 19th century Britain. International Journal of Epidemiology. 2002;31:920-932.
4. Craig P, Cooper C, Gunnell D, Haw S, Lawson K, Macintyre S, Ogilvie D, Petticrew M, Reeves B, Sutton M, Thompson S. Using natural experiments to evaluate population health interventions: new Medical Research Council guidance. Journal of Epidemiology and Community Health. 2012;66(12):1182-1186.
5. Colchero M, Popkin B, Rivera J, Ng S. Beverage purchases from stores in Mexico under the excise tax on sugar sweetened beverages: observational study. BMJ. 2016;352:h6704.
6. Taillie LS, Ng SW, Popkin BM. Gains Made By Walmart’s Healthier Food Initiative Mirror Preexisting Trends. Health Affairs (Millwood). November 1, 2015 2015;34(11):1869-1876.
7. Ng SW, Slining MM, Popkin BM. The Healthy Weight Commitment Foundation Pledge: Calories Sold from U.S. Consumer Packaged Goods, 2007–2012. American Journal of Preventive Medicine. 10// 2014;47(4):508-519.
8. Allison P. Fixed effects regression analysis for longitudinal data using SAS. Cary, North Carolina: SAS Institute Inc;2005.
9. Gunasekara F, Richardson K, Carter K, Blakely T. Fixed effects analysis of repeated measures data. International Journal of Epidemiology. 2014;43(1):264-269.
10. Wooldridge J. Introductory econometrics: a modern approach. 3rd ed. Mason: Thomson South-Western; 2006.
