# Supplementary material for: The impact of voluntary front-of-pack nutrition labelling on packaged food reformulation: A difference-in-differences analysis of the Australasian Health Star Rating scheme
Source: PLoS Med. 2020 Nov 20;17(11):e1003427. doi: 10.1371/journal.pmed.1003427 (PMC7679009; doi:10.1371/journal.pmed.1003427)
Supplement: Attachment — Submitted filename: PLOSMed_REV_letter_to_editor_19May2020.pdf [file pmed.1003427.s010.pdf]

The Editorial Board,

PLOS Medicine

May 20, 2020

Dear Member of the Editorial Board,

RE: PMEDICINE-D-20-00808R1 - The Impact of Voluntary Front of Pack Nutrition Labelling on Packaged Food Reformulation: A difference-in-differences analysis of the Australasian Health Star Rating scheme

Thank you for your consideration of our manuscript at your journal, and for the referee comments. We feel our incorporation of changes responding to the comments have greatly improved the manuscript. Please find our responses to the comments received below. Changes to the manuscript are highlighted in red.

Sincerely

Laxman Bablani (and on behalf of co-authors)

S.No	Context	Comment	Author Response	Page# of edit in resubmitted manuscript
**Editorial Comments**				
1	Methodology	We'd ask that you clarify in the paper if the analytical approach reported in the paper was planned out prospectively. Please state this (either way) in the Methods section.<br><br>a) If there was a prospective analysis plan (ie from the your funding proposal/protocol) used in designing the study, please include the relevant prospectively written document with your revised manuscript as a Supporting Information file to be published alongside your study, and cite it in the Methods section. A legend for this file should be included at the end of your manuscript.<br><br>b) If there was no such prospective analysis plan, please make sure that the Methods section transparently describes when analyses were planned, and when/why any data-driven changes to analyses took place.<br><br>c) In either case, changes in the analysis-- including those made in response to peer review comments-- should be identified as such in the Methods section of the paper, with rationale.	A short note was added to the methods section:<br><br>This was an investigator-initiated study funded by a Health Research Council of New Zealand programme grant (18/672), and the grant application broadly specifies the research design. This study conforms to the broad research design and questions therein. Data available in late 2019 was used to provide timely evidence for the program. The nutrient profile score was replaced with HSR score as an outcome to enhance the study's relevance, since most stakeholders observe the HSR score. Portions of the grant relevant to this study are provided in Appendix S9.	5
2	Abstract	In the last sentence of the Abstract Methods and Findings section, please describe the main limitation(s) of the study's methodology.	Although confounding is a major issue with observational studies, we are confident that the use of econometric techniques has been able to establish the causal reformulation effect of HSR at a SKU level. However, results are not sales weighted. Based on reviewer comments (see #16), we felt this was an important limitation.<br><br>The following text was, therefore, added to the abstract:<br>A limitation of our study is that results are not sales-weighted. Thus, it is not able to assess	2

			changes in the overall food sample that occur because of HSR-caused reformulation.	
**3**	Limitations	In the Limitations section of the Discussion, this mentions the parallel path assumption as being a key issue, which if violated could be a major limitation of the study. Many readers may not know what a parallel path assumption is, and for more general readers (for whom the topic matter may be of great interest, even if they don't understand the specialist methods used here), it would be good to say something briefly about what that assumption is and how/why it is relevant to such methodological approaches as used here.	A short note was added:<br><br>*Further, differences-in-differences estimates rely on the parallel path assumption* – reformulation amongst HSR and non-HSR products would have been comparable, had HSR not been introduced.	17
**4**	Methodology	I noted that the STROBE reporting tool is used and supplied as a checklist in the supporting files. We'd recommend that you add a brief sentence in the Methods to note that STROBE was used in reporting the study.	A sentence confirming that the study conforms to STROBE guidelines was added to the "Study overview" subsection.<br><br>Reporting of this study conforms to STROBE guidelines (15) (Appendix S8).	5
		**Reviewer 1**		
**5**	Discussion	I am not a content expert in this area, so I couldn't comment on the findings implications for Australia and New Zealand public health policy. If I were however to nitpick, I wonder how generalizable the findings are to other countries?	The comparison with previous studies was altered to make the comparison clearer. See also: #15.<br><br>FoPL systems include a wide range of designs and policy, and public health authorities are faced with a wide range of effect sizes across various schemes and observation study designs. Our study produces results that are more muted than those arising from the Dutch choices logo program(8) which highlights positive nutrients, however this may reflect differences in study design. To the best of our knowledge, no studies have causally assessed the reformulation effect of many widely used or cited FoPL schemes such as traffic light signals highlighting levels of individual nutrients in the UK, the summary graded Nutri-score in the EU, or warning logos for sodium, energy, sugars, and saturated fats in Chile. Despite the absence of such studies, our	16

			results shall serve as a close benchmark for similar graded summary schemes, such as Nutri-score (22), which has seen increasing uptake across Europe.	
**Reviewer 2**				
**6**	Introduction	An explanation of how FOPL is expected to encourage reformulation is needed between the 1st and the 2nd paragraph	The following text was added to the introduction:<br><br>Such labels are designed to allow consumers to discern healthier items more effectively than descriptive back of package labels (1). Consumers are more likely to choose products they perceive to be healthier (2). The expectation of labelling influencing consumer choice towards healthier products, and subsequently affecting industry profits, can encourage industry-led reformulation of packaged food products.	4
**7**	Introduction	The authors should include references to the effects of the Chilean food law on food reformulation.	The reformulation effects of the Chilean food law have not been clearly identified, to the best of the authors' knowledge.<br><br>We have, therefore, added the following text to the introduction:<br>Analyses of Chile's food labelling laws found limited anticipatory reformulation (9), with a subsequent study (10) finding large changes in energy consumption arising from sugar sweetened beverages (11.9 kcal/capita/day [95% CI: −12.0 to −11.9], but being unable to split out changes due to industry-led reformulation or consumer behaviour changes.	4
**8**	Methodology	The databases should be more clearly explained, i.e. what type of information is available in the database. The authors should better explain how comparable the NZ and Australian data are.	The following text was added to the Methods section:<br><br>Both photographic surveys present largely comparable information on packaged food products sold in each country. Each contains SKU codes, and brand and product identifiers.	6

			They also contain data on all nutrients mandatorily listed on the NIP – energy, sodium, sugar, saturated fat, and protein. Additional nutrients and micronutrients, such as fibre, vitamins, or minerals are also captured if listed on the NIP. The presence of front of pack labels, such as HSR and the actual HSR score is contained within each dataset. Each dataset also performs an imputation of HSR across products, using ingredient information to calculate the Fruit, Vegetable, Nut, and Legume (FNVL) content scores.	
**9**	Results	For the products that were reformulated, do the authors have information about how they were reformulated? I.e., was sugar reduction achieved by adding sweeteners? was salt-reduction achieved by adding KCl? This information is highly relevant to fully understand the potential effects of reformulation on intake of additives and also on changes in the sensory characteristics of products that could encourage changes in food preferences. This should also be included in the discussion.	We thank the reviewer for this comment and, although it is beyond the scope of this paper, agree that it presents an important opportunity for future research.<br><br>Our changes in response to this comment overlap with #12.<br><br>We have added the following text to the discussion:<br><br>Although our results show that HSR labelling is associated with reformulation, we did not establish changes in nutrient composition or additives that underpin such reformulation. Such changes, e.g. adding artificial sweeteners, have important consequences for both the health implications (28) and sensory characteristics (29) of the products studied. A lower-level analysis of the changes to ingredients that affect product reformulation and consequences thereof, although beyond the scope of this study, is an important avenue for future research. There is also a growing literature that highlights the health concerns of consuming ultra-processed foods in general (30, 31). An analysis of policies that improve population diets through increasing	17-18

			consumption of unprocessed and minimally processed foods presents a largely unaddressed vital area for future work.	
**10**	Results	Can the authors separate product reformulation and product innovation (i.e. new healthful products entering the marketplace)? If not, they should discuss this as a limitation and additional potential effect of FOPL.	A note addressing this limitation was added to the paper.<br><br>Further, this study focusses on product reformulation effects. Healthier product innovation, where new healthful products enter the marketplace, is another potential effect of HSR. However, the selection of already healthy products into HSR and the absence of a comparator group for such newly innovated products mean that these innovation effects could not be estimated by this study.	17
**11**	Discussion	The discussion should include a critical overview of the HSR given that it is mainly used in the more healthful products. The authors state that results suggest that the use of HSR should be incentivized. However, results from the study suggest that it should be made compulsory	We agree with the reviewer.<br><br>The first paragraph of the implications is now:<br>Although market-based mechanisms may also cause near universal adoption of such schemes in theory (26), such adoption is rarely observed in real-life (27), or even the analysis sample used here. Mandatory adoption of FoPL for less healthy products is likely to maximise the public health gains arising from reformulation, and likely also from changes in consumer behaviour.<br><br>The conclusion was changed to:<br>To maximise the reformulation effects of voluntary FoPL, governments need to make such schemes mandatory.	17,18

**12**	Discussion	The authors should include an in-depth discussion of the limitations of product reformulation in the light of the growing body of evidence associating ultra-processed products with NCDs, regardless of their nutritional composition.	Our response to this comment overlaps with #9. The following text was added to the implications:<br><br>Although our results show that HSR labelling is associated with reformulation, we did not establish changes in nutrient composition or additives that underpin such reformulation. Such changes, e.g. adding artificial sweeteners, have important consequences for both the health implications (26) and sensory characteristics (27) of the products studied. A lower-level analysis of the changes to ingredients that affect product reformulation and consequences thereof, although beyond the scope of this study, is an important avenue for future research. There is also a growing literature that highlights the health concerns of consuming ultra-processed foods in general (28, 29). An analysis of policies that improve population diets through increasing consumption of unprocessed and minimally processed foods presents a largely unaddressed vital area for future work.	17-18
		**Reviewer 3**		
**13**	Introduction	The report seems to be focused importantly on readers from Australia or New Zealand who are well aware of the system. This is evident at different levels. In order to properly reach a broader spectrum of readers around the world, I suggest to present a picture of the FOPL (it may be added as supplementary material).	Including the logo would have made the context clearer<br><br>Unfortunately, PLOS Medicine could not include the logos in the main paper or supplements, even with permission from the copyright holder.<br><br>They conflict with the CC-BY-4.0 license used for open access publishing by PLOS Medicine.	
**14**	Introduction	Also, it should be indicated the overall usage of the FOPL in each country earlier in the text, currently this information is only reported at the end of the manuscript.	The following clarification was added to the introduction:	4

			Since its introduction, HSR has seen reasonable acceptance, and was displayed on about 23% of NZ products in 2019, and 31% of Australian products in 2018 (Appendix S1 graphs the percentage of foods using HSR across years in Australia and NZ).	
**15**	Discussion	In the same line, the report should aim to inform local health authorities and readers, but also be helpful to other readers form other countries who might be discussing which FOPL schemes to implement. In this regard, the discussion should expand the comparison of the reported result s with the ones reported with other FOPL schemes.	Our response to this comment overlaps with #5.<br><br>There are few relevant results for other FoPL schemes. The following changes were made to the comparison with other studies section:<br><br>FoPL systems include a wide range of designs and policy, and public health authorities are faced with a wide range of effect sizes across various schemes and observation study designs. Our study produces results that are more muted than those arising from the Dutch choices logo program(8) which highlights positive nutrients, however this may reflect differences in study design. To the best of our knowledge, no studies have causally assessed the reformulation effect of many widely used or cited FoPL schemes such as traffic light signals highlighting levels of individual nutrients in the UK, the summary graded Nutri-score in the EU, or warning logos for sodium, energy, sugars, and saturated fats in Chile. Despite the absence of such studies, our results shall serve as a close benchmark for similar graded summary schemes, such as Nutri-score (22), which has seen increasing uptake across Europe.	16

**16**	Discussion/Methods	More importantly, in order to increase the relevance of the results, I strongly suggest including in the analyses the overall reformulation of the analytical sample. For example, how meaningful is the decrease in 42 mg of sodium per 100g/ml for labeled foods in the context of the partial use of the label? How much has the overall food supply (or the analytical sample) improved? That information is surely more helpful for informing decisions (for instance the need to implement mandatory labels) both locally and in other countries (even understanding that a difference-in-difference analysis would not be possible).	The following changes to text were made in the limitations subsection:<br><br>A limitation of our study is that results are not sales-weighted. Thus, it is not able to assess changes in the overall food sample that occur because of HSR-caused reformulation. This limitation in the study design was motivated by the fact that sales weights are also affected by HSR - for instance, the consumption of less healthy products may decrease post labelling which further affects food supply. An analysis of the overall food supply must include both changes in consumer and industry behavior. This is outside the scope of the study and its datasets, and we aim to address it separately. However, the modest results herein suggest that overall changes to food supply due to reformulation caused by HSR are likely to be limited. Making HSR mandatory is likely to improve the healthfulness of consumer diets by causing more less healthy products to adopt the label.	15-16
**17**	Discussion	Besides what said under 'General Comments', authors could further discuss on the implications of the results. For instance, they quote a previous report modeling the impact of the reported reformulation on DALYs, without indicating the extend of the reformulation reported in that specific study.	The text was altered to include the effect size in the implications section:<br><br>*the previous Australian study,* which found an energy reduction of -7.11 kJ/100g, *also estimated the health impact of HSR using simulation models*	17
**18**	Discussion	Moreover, regarding food technology and processing of food, further discussion could be done on the addition of fiber to food, ingredients that could replace sugars or sodium on foods and beverages, or the concern regarding processing itself.	We felt this comment addressed similar concerns as comment #9 above.<br><br>*Please see our response above.*	17-18
**19**	Discussion	When expanding the discussion to the effect of other FOPL schemes on reformulation, differences between such schemes could be also discussed (the ones focused on positive aspects vs the ones focusing in negative aspects, etc).	As highlighted in #15, we highlight the lack of comparable studies (or any study) for most other FoPL. Some features of each scheme are	

			highlighted, but a complete discussion shall not be suitable for this paper.<br><br>Separately (for instance, #16; #11), we highlight mandatory adoption of FoPL is likely to cause the highest public health impact.	
**20**	Results	In order to better understand the dynamics of the HSR implementation, I suggest adding a graph showing the percentage of foods using the label every year (I imagine the current use was achieved after several years of implementation).	The graph in Appendix S1 addresses this comment. The following line was added to the introduction:<br><br>(Appendix S1 graphs the percentage of foods using HSR across years in Australia and NZ).	Pg. 4
**21**	Results	Table 1 and 2 display symbols to represent p-values, however such symbols are not used in the tables.	We thank the reviewer for noting this error. The references to the symbols have been removed.	Pg. 12, 14
**22**	Methods	How was handled the nutrition information of products needing reconstitution (i.e. powder or concentrated soups or fruit drinks)?	Both datasets contain details of reconstituted food using internally consistent, but not identical methods. We have added the following text to the main paper:<br><br>Both datasets differently treat NIPs for foods are prepared (say, dry soup mix); within each dataset, such NIPs are treated consistently. Nutritrack reports “as-sold” NIPs by default; FoodSwitch reports “as-prepared” NIPs. The small number of products affected (<5%), consistent treatment of NIPs within datasets, and use of fixed effects methods ameliorates much of the impact of such differences. S2 Appendix provides more detail on the handling of “as-prepared” NIPs<br><br>Further details are in S2 Appendix:<br><br>**FoodSwitch (Australia)**<br>FoodSwitch (AU) tracks whether NIPs are reported as “As-sold” or “As-prepared” when more than one NIP is reported on the product. Only 2.0% of products reported more than one	Pg 6, S2 Appendix

			NIP. For products that report only one NIP, the NIP reported on the packet is used, but the type could not be identified by us. In cases with ≥ 2 NIPs, the "as-prepared" NIP is used for consistency across types. The top 5 food groups with multiple NIPs are sauces (includes gravies), breakfast cereals, cake mixes, pasta, coffee & tea, and rice.<br>Consistent coding within FoodSwitch prevents biases in the coefficients due to the fixed effects analyses from inconsistent NIP information. Further, standard errors are smaller due to such consistent coding.<br><br>Nutritrack (New Zealand)<br>A variable in the Nutritrack (NZ) dataset indicates if a product Nutrition Information Panel (NIP) reports the "as sold" product composition or the composition when reconstituted. The "as sold" composition data are the default in the database unless only reconstituted composition is reported on a product NIP. 4.6% of product NIPs report reconstituted composition.<br>Of these foods, most are reconstituted with water. The top 10 foods groups with reconstituted nutrition data are: Dry soup mix, flavoured noodles, coffee, gravies or stocks, cordials, yogurt dry mix, tea, pasta, and cake mixes. Thus >95% product nutrition information used in our analyses was for non-reconstituted products. Our results for NZ are largely unchanged when removing such reconstituted foods.<br><br>In both cases, consistent coding across datasets prevents biases arising in our fixed effects analyses from inconsinstent NIP information.	

**23**	Methods	I understand authors decided to use the imputed scores and I agree with the decision, but I suggest to include a sensitivity analysis grouping products based on the actual use of the FOPL, assessing just the nutrient content and energy density (given the HSR score will not be possible to evaluate).	The exposure already corresponds to the *actual* use of HSR labelling by products, although HSR scores are imputed to provide a consistent outcome that is most directly targeted by the policy.<br><br>In our understanding, therefore, the analysis already mirrors this suggested analysis for the six nutrients studied in tables 2 and 3.	
**24**	Methods	Sensitivity analyses aim to validate in different way the counterfactual; one of such considers the reformulation trend before the implementation of the measure. How was that possible in the case of Australia, where only one time point was collected before the implementation?	We note the time used for this analysis is relative to the point of labelling with HSR. Products undertook labelling at different years from 2015 onwards, and therefore we can observe several time points before reformulation for a product that underwent reformulation in, say, 2018.<br><br>Changes in reformulation, due to differences between groups or anticipatory effects, etc., would cause this trend to be statistically significant, but little evidence was found.<br><br>Further details and the regression specification are presented in Appendix S4. We believe no changes to the text are needed in this case.	
**25**	Methods	The sample could be better described, specifying food groups considered, and the relative proportion of them included in each group (labeled vs unlabeled products). I suppose authors did not provide results by food group given the magnitude of the results. However, understanding the food group composition of the sample and specially of the labeled and unlabeled groups is key to better understand and interpret the results. Differences in the relative proportion of food groups may be influencing some observations. I understand that the sensitivity analyses balancing groups by pre-labelling nutrient information is aiming to address this issue, but matching by nutrient characteristics is not the same that matching by food groups. Reformulation may be easier in a given food group due to technological characteristics of that particular food group.	A description of the sample, in terms of food groups, presented as a supplementary material, in appendix S1.<br><br>The following text was added to the results section:<br>Appendix S1 presents the number of observations by food groups for products that never adopted HSR, and those that adopted HSR across our study period, and the last year of observation (2018 for Australia, 2019 for NZ). Adoption in both countries is led by cereals (51% in Australia 2018, 36% in 2019 for NZ) and convenience foods (45% in Australia	9

			2018, 37% in 2019 for NZ). Processed meat, fish, fruit, and vegetable products also saw greater than average adoption.<br>Product- and food group-level nutrient composition in the two countries affected reformulation profiles. however, the choice of comparator group largely does not affect the results.<br><br>Further, we initially considered analysing reformulation patterns within food groups – individual food groups reformulate in an idiosyncratic manner, and it is difficult to highlight clear patterns. Smaller sample sizes are also likely to increase false positives for many food groups. We do not present results for individual food groups due to such issues.	
**26**	Methods	On the other hand, even if the results are not sale-weighted, authors should provide more information about how relevant are the<br>products included (any information on either market or dietary share is needed). Currently, author provides information about the relevance of supermarket, but something similar should made available at the level of products.	The product-level relevance is likely to be similar to supermarket-level relevance.<br><br>We added the following line to the data sources subsection:<br>Over 2013-2019, the Nutritrack products surveyed accounted for 81.5% of purchases recorded in HomeScan NZ, a large consumer survey (excluding fresh food and alcohol purchases). Consumer panel information for Australia was not available.	6
