# Supplementary material for: The impact of voluntary front-of-pack nutrition labelling on packaged food reformulation: A difference-in-differences analysis of the Australasian Health Star Rating scheme
Source: PLoS Med. 2020 Nov 20;17(11):e1003427. doi: 10.1371/journal.pmed.1003427 (PMC7679009; doi:10.1371/journal.pmed.1003427)
Supplement: Attachment — Submitted filename: PLOSMed_REV2_Lettertoeditor_02Jul.pdf [file pmed.1003427.s011.pdf]

The Editorial Board,

PLOS Medicine

23 July 2020

Dear Member of the Editorial Board,

RE: PMEDICINE-D-20-00808R1 - The Impact of Voluntary Front of Pack Nutrition Labelling on Packaged Food Reformulation: A difference-in-differences analysis of the Australasian Health Star Rating scheme

Thank you for agreeing to review a further revised version of our manuscript. We would also like to offer our gratitude to the editorial staff and anonymous referees for ensuring timely peer review, especially during the current disruptive period.

Our responses to the comments are below, and changes to the manuscript are highlighted in red.

Sincerely,

Laxman Bablani (and on behalf of co-authors)

S.No.	Context	Comment	Author Response	Page#
**Editorial Comments**				
**1**	Supplement: STROBE Checklist	1- Thank you for providing your STROBE checklist. Please replace the page numbers with paragraph numbers per section (e.g. "Methods, paragraph 1"), since the page numbers of the final published paper may be different from the page numbers in the current manuscript.	Thanks, done.	S8 Appendix
**2**	Supplement: Grant Application	2- Thank you for providing your grant application as a supplementary text. I notice you redacted portions, and the pages are labeled "IN CONFIDENCE". Please confirm that you are comfortable with this document being published alongside your manuscript.	Thank you for your comment. We confirm that the document may be published as a supplement alongside the manuscript.	S9 Appendix
**3**	Methods	3- Additionally, please note in the methods section any analyses that differ from those that were planned, and provide transparent explanations for differences that affect the reliability of the study's results. For example, if a reported analysis was performed in response to a reviewer's request, please note this. If an analysis was based on an interesting but unanticipated pattern in the data, please be clear that the analysis was data-driven. If hypotheses that were not included in the original study design later became important to test because new evidence became available from other studies, please explain the situation, so that it is clear whether new analyses were data driven or added for another reason.	The following note was added to the methods section, clarifying the reasons for changed analyses:<br><br>Although a prospective design for this study does not exist, it is an investigator-initiated study funded by a Health Research Council of New Zealand programme grant (18/672). Portions of the grant relevant to this study are provided in Appendix S9. The grant specified using fixed effects and differences-in-differences methods to study the impact of HSR on product reformulation, and this study conforms to the broad research design and questions therein. We note the following key changes from the grant: First, data available in late 2019 was used to provide timely evidence for the program. Second, the nutrient profile score was replaced with HSR score as an outcome to enhance the study's relevance, since most stakeholders only observe the HSR score. Data-driven changes to the analysis include dropping a detailed analysis of	Methods -> Study Overview<br><br>(Pg6, Line 198-215)

			FNVL composition across products, due to proprietary algorithms used in imputing FNVL content. We also became aware of issues with fibre content within the datasets, and ran analyses for robustness, as described below. Last, our reviewers provided many valuable suggestions for analyses to improve the clarity of our data sample. These include the addition of all analyses in Appendix S1 and S2, as well as the refinement of the CEM weights used in Appendix S5 to include food group information.	
**4**	Abstract	4- In the Abstract Conclusions, please address the study implications without overreaching what can be concluded from the data; the phrase "In this study, we observed ..." and "Our results suggest…" may be useful.	The conclusion was changed to avoid overreaching:<br><br>In this study, we observed that ~~R~~reformulation effects in response to voluntary HSR labelling are small, but greater for initially less-healthy products. Initially less healthy foods ~~are~~ were however less likely to adopt HSR. Our results, therefore, suggest that ~~M~~mandatory labelling~~, therefore,~~ has the greatest potential for improving the healthiness of packaged foods.	Pg2, line 63-67
**5**	Discussion	5- Please edit the Discussion Conclusions similarly. "*In this setting, we found that* FoPL schemes such as HSR *may* play…" and "To maximise the reformulation effects of FoPL, *we suggest* governments make such schemes mandatory." would be appropriate.	The proposed wording is appropriate, and the conclusion was changed accordingly.<br><br>In this setting, we found that FoPL schemes such as HSR may play a modest role in driving healthier product reformulation, and such reformulation is higher for the least healthy products. The low uptake of HSR overall, and an even lower rate of labelling for unhealthy products limits reformulation. To maximise the reformulation effects of FoPL, we suggest governments ~~need to~~should make such schemes mandatory	Conclusion<br><br>Pg 18, Line 562-567
**6**	General	6- Throughout the manuscript, where relevant, please include p-values alongside 95% CIs	*p*-values have been added when appropriate.<br><br>The notable exception is the *plain language author summary.*	

Reviewer 3				
**7**	General: Sales-weighted data	The abstract state: "We studied the impact of voluntary adoption of HSR on food reformulation overall, and for more- versus less-healthy foods". To me, this means the manuscript will report the reformulation of all available foods after the voluntary adoption of HSR, which is not the case given it reports the reformulation of labeled foods compared to unlabeled foods. As commented in my previous review, I do think it is important to additionally present data on the changes of food composition for the overall food supply collected in both countries, even with no counterfactual, a simple pre-post analysis, to have a sense of the overall impact. Author's response to that comment is that results are not sales-weighted.<br><br>Sales-weighted data would allow providing a greater relevance to reformulation of foods that have a greater market share, compared to reformulation of foods that have little participation in the market. I agree this is very relevant and therefore a limitation of<br>the study. However, sales-weighted data are not needed to evaluate the impact of reformulation on the food supply (i.e., for the purpose of this study, non-seasonal packaged foods available at the main supermarkets during the years of data collection).<br><br>If authors decline to include this extra analysis, please remove the sentence of the abstract, which I think may be confusing for other readers that, as me, could expect to see actual data on the impact of HSR on food reformulation overall.	Thank you for your comment. We apologise for any confusion caused by the wording.<br><br>However, the key research question of the paper remains examining the causal effect of HSR in a DiD setting, which necessitates comparisons between labelled and unlabeled foods. We have therefore made the following changes:<br><br>• The abstract has been changed to: We studied the impact of voluntary adoption of HSR on ~~overall~~ food reformulation relative to unlabelled foods and examined differential impacts for more- versus less-healthy foods.<br>• RE: Sales weighted data: it remains an important limitation, although we have changed the wording to avoid confusion. See below.<br>• A pre-post analysis of the overall sample in the lack of an intervention is sensitive to many assumptions. In lieu, a descriptive analysis of the trends in nutrient composition for the overall sample in each year (Nutritrack: 2013-2019; FoodSwitch: 2014-2018) has been added to Appendix S1; and the following text was added to the “Results” section:<br><br>Last, Appendix S1 also graphs the overall trends in nutrient composition across the datasets in the study period showing, for instance, energy density in the NZ sample increases from 1095 to 1134 kj/100g or ml; while the energy density of the Australian sample decreases slightly from 1117 to 1104 kj/100g or ml. Such underlying trends in overall sample	

			composition highlight the reasons for using year and product fixed effects in our analysis, as they may confound analyses for the causal effect of HSR.	
**8**	Abstract	Please revise the following statement from the abstract states: "A limitation of our study is that results are not sales-weighted. Thus, it is not able to assess changes in the overall food supply that occur because of HSR-caused reformulation." As previously commented, the fact that results are not sales-weighted does not mean that authors cannot assess changes in the overall food supply. I might be missing something, in that case please provide an explanation for other readers as me who do not see the connection.	Thank you, the statement has been revised to:<br><br>A limitation of our study is that results are not sales-weighted. Thus, it is not able to assess changes in overall nutrient consumption that occur because of HSR-caused reformulation.	Abstract -> Methods and Findings<br><br>(Pg2, Line 60)<br><br>Author Summary (Pg 3, line 109)
**9**	Author Summary	In author summary, please consider rephrasing this statement "Initially unhealthy products increase their HSR rating by more than 0.1 stars, while healthier products show less reformulation - a 1 star increase in initial healthiness reduces reformulation by around 0.04 stars."	To clarify the language further, it has been changed to:<br><br>Initially unhealthy products that adopt HSR increase their rating by more than 0.1 stars. This effect becomes smaller the greater the initial healthiness of the product– a 1 star increase in initial healthiness reduces reformulation by around 0.04 stars.	Pg2,<br><br>lines 101-103
**10**	Introduction	Regarding the use of HSR label, the introduction states: "Since its introduction, HSR has seen reasonable acceptance, and was displayed on about 23% of NZ products in 2019, and 31% of Australian products in 2018 (Appendix S1 graphs the percentage of foods using HSR across years in Australia and NZ)." Can you please rephrase clearly indicating the percentage of use has been continuously increasing since the adoption of the policy until reaching those percentages in 2019 (to better reflect what is seen in S1 graphs).	The text has been changed to:<br><br>Since its introduction, HSR has seen ~~reasonable~~ steadily increasing acceptance, and was displayed on about 23% of NZ products in 2019, and 31% of Australian products in 2018 (Appendix S1 graphs the percentage of foods using HSR across years in Australia and NZ).	Pg 4, Line 124

**11**	Results: Table 1	Moreover, from table 1 one could interpret the HSR label adoption was about 6% in New Zealand (1785 unique products out of 28053) and 8.5% in Australia (2462 unique products out of 26605). Please provide an explanation for those differences? Are they explained by the gradual adoption of the label? Was there a lower adoption rate within the sample you collected?	Table 1 describes nutrient information for products that never displayed HSR, and products which adopted HSR*, in the year before labelling*:<br><br>The proportion here is not the same as adoption because:<br>• Newly innovated HSR adopting products are not included, since there is no observation in the year before labelling.<br>• Similarly, there are many products that had not adopted HSR and are removed from supermarkets for various reasons. There are generally 13-16,000 products each year (e.g. table in S1 appendix for 2018-2019)<br>• The fixed effects analysis accounts for many sample composition issues by requiring a product to be observed in at least two years to contribute to the reformulation time trend, since it controls for the mean. It also looks at differences *within* products on adoption of HSR – requiring at least one observation before HSR is implemented, and one observation after to determine the causal effect.	
**12**	Methods	Another concern I have is the different composition (regarding food groups) of the labeled subset and the counterfactual unlabeled subset. As shown in S1 table, compared to never labeled products, cereal products are 2 times more frequent within the subset of foods adopting the label (8.8 vs 16.7%, respectively), whereas sugars and related products are 4 times less present in that group (2.5% vs 0.6%, respectively). Those different food group distributions may be explained by differences in the technological feasibility for reformulation between food groups. Thus, I think a bold sensitivity analysis would be something similar to the first one	Differences in food group composition between the treated and comparison group may bias estimates from our observational study.<br><br>The CEM matching exercise was updated to match within the major food groups the products belonged to, and the results remained robust to the change.<br><br>Please see Supplement 5.<br><br>Further, the manuscript text has been updated to:	Methods > Analysis > Sensitivity Analyses<br><br>(Pg 8, Line 323)<br><br>*And*<br><br>S5 Appendix

		considered (i.e., coarsened exact matching (CEM) is a non-parametric matching technique that balances pre-labelling nutrient information between HSR products and products that never received HSR labelling), but balancing pre-labelling food groups classification between HSR products and products that never received HSR.	First, coarsened exact matching (CEM) is a non-parametric matching technique that balances pre-labelling nutrient and major food group information between HSR products and products that never received HSR labelling (20).	
**13**	Results: Table 1	Moreover, I suggest S1 table displays percentages of food groups considering the subset (i.e., never HSR labelled vs adopted HSR) as denominator, in order to clearly see the difference in food groups composition between both subsets.	Thank you, the suggested change does enhance the understanding of differences in food group composition between subsets. Table 1 in S1 has been updated accordingly.<br><br>The relevant lines in the manuscript have been changed to:<br>Adoption in both countries is led by cereals, convenience foods, processed meat, fish, fruit, and vegetable products.	S1 Appendix > Table 1<br><br>Results (Pg9, Line 344-345)
**14**	Results	Results section: Values for sodium and sugars in the text are different than the ones displayed in Table 2.	Thank you, fixed.	
**15**	Discussion	First paragraph of the discussion should include the prevalence of labelled products in order to better interpret the magnitude of those changes on the overall food supply (or provide information on the extra analysis proposed, if considered).	The following revision was made:<br>However, only 23% and 31% of products in New Zealand (2019) and Australia (2018) respectively had adopted HSR. The effect of mandatory labelling may, therefore, not be a linear extrapolation from partial uptake due to the voluntary nature of many FoPL schemes	Discussion<br><br>Pg 15, lines 467 - 469
**16**	Discussion	Regarding the limitation that results are not sales-weighted, and thus authors are not able to assess changes in the overall food sample that occur because of HSR-caused reformulation, please considered my earlier comment made regarding the same issue	The statement has been revised to:<br><br>An important limitation of our study is that results are not sales-weighted. Thus, it is not able to assess changes in overall nutrient consumption that occur because of HSR-caused reformulation. This limitation in the study design was motivated by the fact that sales weights are also affected by HSR - for instance, the demand for less healthy products may decrease post	Discussion -> Strengths and Limitations<br><br>(pg 16, lines 498-509)

			labelling which further affects consumption. An analysis of the consumption effects of dietary policy must include both changes in consumer and industry behavior. This is outside the scope of the study and its datasets, and we aim to address it separately. However, the modest results herein suggest that overall changes to nutrient consumption due to reformulation caused by HSR are likely to be limited. Making HSR mandatory is likely to improve the healthfulness of consumer diets by causing more less healthy products to adopt the label.	
**17**	Discussion	Although I understand why the sentence "There is also a growing literature that highlights the health concerns of consuming ultra-processed foods in general" was added, please consider including "despite the content of healthy and unhealthy nutrients of such foods" or something similar. As it is right now, I do not think the idea will be clear for all readers.	To improve clarity, the line was changed to: Irrespective of the density of healthy and unhealthy nutrients in food, ~~T~~there is also a growing literature that highlights the health concerns of consuming ultra-processed foods in general (30, 31).	Discussion -> Implications<br><br>Pg 18, Line 547-549
**18**	General	"NIP" is defined several times within the text.	Thank you, fixed	
**19**	Supplement: S2	S2: Please describe what Nutrition Information Panel (NIP) stand for earlier in the text.	The abbreviation NIP has been expanded upon in the heading	S2 Appendix
**20**	Supplement: S3	S3: Title reads "S3 Appendix: Tables underlying Figure 4,5" I imagine it should read "S3 Appendix: Tables underlying Figure 4". Please be consistent with headings and order of columns between S3 and tables 2 and 3.	The appendix has been re-titled: S3 Appendix: Tables underlying Figure 3,4.<br><br>Headings and the order of columns have been harmonized between S3 and Table 2.	S3 Appendix
