# Supplementary material for: The impact of voluntary front-of-pack nutrition labelling on packaged food reformulation: A difference-in-differences analysis of the Australasian Health Star Rating scheme
Source: PLoS Med. 2020 Nov 20;17(11):e1003427. doi: 10.1371/journal.pmed.1003427 (PMC7679009; doi:10.1371/journal.pmed.1003427)
Supplement: Attachment — Submitted filename: PLOSMed_REV_letter_to_editor_07Oct.docx [file pmed.1003427.s012.docx]

The Editorial Board,

PLOS Medicine

Tuesday, 06 October 2020

Dear Member of the Editorial Board,

RE: PMEDICINE-D-20-00808R3- The Impact of Voluntary Front of Pack Nutrition Labelling on Packaged Food Reformulation: A difference-in-differences analysis of the Australasian Health Star Rating scheme

Thank you for your consideration of our manuscript at your journal, and for further comments. We thank the staff and three anonymous referees for providing timely peer review throughout these strange times.

We are delighted that our paper has been provisionally accepted. Please find below a checklist of issues raised by editorial and production staff, and our responses below. Changes to the manuscript are highlighted in red.

Sincerely,

Laxman Bablani (and on behalf of co-authors)

| **S.No** | **Context** | **Comment** | **Author Response** | **Page#/reference for edit** |
| --- | --- | --- | --- | --- |
| **Editorial Comments** | | | | |
| **1** |  | Please edit the data statement to read:  “Because of commercial and legal restrictions to the use of copyrighted material it is not possible to share data openly which reveal the product or supermarket names, but unredacted versions of the dataset are available with a licensed agreement that they will be restricted to non-commercial use. For access to Nutritrack, please contact the The National Institute for Health Innovation at the University of Auckland at [enquiries@nihi.auckland.ac.nz](mailto:enquiries@nihi.auckland.ac.nz). For access to FoodSwitch, please contact Fraser Taylor, managing director for Foodswitch [ftaylor@georgeinstitute.org.au](mailto:ftaylor@georgeinstitute.org.au) or [foodswitch@georgeinstitute.org.au](mailto:foodswitch@georgeinstitute.org.au).” | Done | Metadata |
| **2** |  | You use both “less healthy” and “unhealthy” foods, please be consistent throughout the manuscript. | Done | Manuscript |
| **3** |  | Early in the Abstract Methods and Findings, please note the number of products in the analysis and specify the classes of product included (cereals, etc). | Added to abstract:  The analysis sample covered 58,905 unique products over 14 major food groups. | Abstract |
| **4** |  | Throughout the manuscript, please check that you are consistent with your use of positive and negative changes when describing declines or increases in content. For example, the Abstract Methods describes: “ Labelled products showed a -4.0% [95% CI -6.4% to -1.7%, p=0.001] relative decline in sodium content in NZ, and there was a 1.4% [95% CI: 2.7% to 0.0%, p=0.045] sodium change in Australia.”, when it seems that both countries experienced a decrease (i.e., should it be -1.4%?). | Done | Manuscript |
| **5** |  | Additionally, please be clear that the difference in sugar content observed in Australia was not significant (you do so in the Author Summary, but not the Abstract). | Done | Abstract |
| **6** |  | The Abstract Methods and Findings could note that you did not find changes in protein or saturated fat content. | Done | Abstract |
| **7** |  | As this study is observational, please avoid language that implies causality (e.g., “impact”, “effect”) throughout. Examples include the last sentence of the Abstract Background, the first sentence of the Abstract Conclusions, the third point of the Author Summary, the end of the Introduction, the opening sentence of the Discussion. | We would like to appeal against such a change.  While the study is observational, it uses several econometric methods including multiple regression, matching, and fixed effects. It also conducts several sensitivity analyses. The aim of these analyses is to derive associations with causal interpretation, and therefore causal language is used ^[1]^ ^[2]^.  Further, several papers in PLOS Medicine have such causal language in an observational setting. For a recent example, see ^[3]^ | Manuscript |
| **8** |  | It may be also worth noting the limitations of the observational design alongside other limitations in the Abstract. | Added the sentence:  Also, participation into labelling and reformulation are jointly determined by producers in this observational study, impacting its generalizability to policies with mandatory labelling. | Abstract |
| **9** |  | The Introduction could include a bit more on the health impacts of healthier diets (e.g., major diseases linked to sodium, sugar, and fiber content). | Introductory sentence changed to:  Population-based approaches are required to combat unhealthy diets which have been linked to several non-communicable diseases, including cardiovascular diseases, diabetes, and cancer (1). | Page 4,  Introduction |
| **10** |  | Thank you for providing p values. Please remove the italics. | Done | Various, Abstract and Manuscript |
| **11** |  | Please also remove the italicized text used for emphasis (e.g., bottom of page 13 “The second row contains the *change* in reformulation when baseline HSR *increases* by 1. ” | Done | Manuscript |
| **12** |  | Bottom of page 13: stray equals sign? (=0.17-0.04) | The equal to sign helps guide readings into understanding the interpretation of the regression. Changed to ‘or’:  0.13 (or, 0.17-0.04) | Page 13.  " Stratification by pre-treatment imputed HSR rating” |
| **13** |  | The Funding and Competing interests statements can be removed from the main text and reside in the metadata fields. | Done. | Metadata |
| **14** |  | Please move the Acknowledgements section to the end of the main text. | Done. | Manuscript |
| **15** |  | Thank you for providing the redacted grant text. We note that this file has a copyright symbol on it. Please excerpt the non-redacted text into a new document and replace S9, noting what has been done in the Supplementary file legend. | Done. | S9 Text |
| Production Issues | | | | |
| **1** | Figures and graphics | Please remove embedded figures from your manuscript file and ensure the most up to date versions of your figures are uploaded to our submission system as separate TIF or EPS files. Please do not remove the figure legends or captions from the manuscript. | Done. | - |
| **2** | Figures and graphics | Please ensure that all main figures are referenced in the manuscript as Fig 1, Fig 2, etc. (including capitalization), rather than Figure 1, Figure 2, etc. Please note, however, that the file names themselves must not include the space, i.e. Fig1.tif, Fig2.tif. Please ensure that all main figure files are clearly labeled as such in the File Inventory. | Done. | - |
| **3** | Metadata | Please ensure only the authors' initials are included in the competing interest statement, not their full names or titles. | Done. | - |
| **4** | Metadata | In accordance with PLOS' data policy, please ensure that your Data Availability Statement in the submission form clearly identifies how readers can access your data. Note that it will be typeset as it is written so please ensure it is in complete sentences and appears as you would like it to in the published version.  For details on our data policy, please see <http://journals.plos.org/plosmedicine/s/data-availability> | Done. | - |
| **5** | Article file | Please indicate the corresponding author on the title page with an asterisk (*) and include the corresponding author's email address. Only an asterisk and the email address itself are required.  Please format as follows, omitting all other name, address and contact information:  * [bablanil@unimelb.edu.au](mailto:bablanil@unimelb.edu.au) | Done. | - |
| **6** | Article file | Please ensure that all authors have at least one affiliation listed on the title page and that all listed affiliations are attributed to at least one author. Affiliations should be listed in ascending numerical order in the affiliation list and should match what is provided in the Authors Information in the submission form.  Affiliations should include an institution, a city, and a country (no postcode). US affiliations need the state as well, written out in full (with no abbreviations). | Done. | - |
| **7** | Article file | Please ensure that all author names are spelled correctly in the manuscript file and the submission form, including the use of a middle name/initials, and any special characters (accents, umlauts, etc.). Please place first/given names before last/family names. We ask that you ensure they match verbatim. Please be aware that correct spelling and formatting of author names in the article file is vital for accurate indexing in PubMed.  The following author names do not match verbatim across the article file and the submission form:  Cliona Ni Mhurchu (Cliona NiMhurchu)  Tony Blakely (Tony BLAKELY) | Done. | - |
| **8** | Article file | We have noted that you have the following present in your manuscript: Financial Disclosure (Funding)/ Competing Interests statement.  We have removed these section from your manuscript file as this information is automatically pulled from the submission form. Please make sure that the information in the submission form is correct. | Done. | - |
| **9** | Supporting information | Please note that you have included reference citations in file S9 Appendix (healthpaper_supplement9.pdf). Since each supporting information file is self-contained, we ask that you please list all cited references in full in that specific file under a 'Reference' heading and start numbering the citations at 1. | Done. | - |
| **10** | Supporting information | Please upload your Supporting Information items individually, rather than in a single file, as you refer to them individually in the text. If you would like to retain the single file please rename internal items (e.g. tables) as A, B, C etc. Please also provide a legend for each under the heading Supporting Information. Supporting Information files should be less than 10 MB each. Please edit any citation of internal items within your Supporting Information files to using letters rather than numbers, e.g. “Table A in S1 Text”. Please ensure these changes are made throughout the manuscript and within the Supporting Information files themselves. | Done. | - |
| **11** | Supporting information | Please ensure that all Supporting Information files use the following format verbatim throughout the manuscript (including legends and all in-text citations): S1 Fig, S1 Table, S1 Text, S1 Data, etc. Each file type should start at 1 and increase numerically. Please note, however, that the file names themselves must use an underscore rather than a space, i.e. S1_Fig.tif, S1_Table.xlsx, S1_Text.doc, S1_Data.xlsx, S1_PRISMA_Checklist.docx, etc. | Done. | - |

1. Miguel A. Hernán, ‘The C-Word: Scientific Euphemisms Do Not Improve Causal Inference From Observational Data’, *American Journal of Public Health* 108, no. 5 (22 March 2018): 616–19, https://doi.org/10.2105/AJPH.2018.304337.
2. Jay S. Kaufman, ‘Commentary: Causal Inference for Social Exposures’, *Annual Review of Public Health* 40, no. 1 (April 2019): 7–21, https://doi.org/10.1146/annurev-publhealth-040218-043735.
3. Peter Scarborough et al., ‘Impact of the Announcement and Implementation of the UK Soft Drinks Industry Levy on Sugar Content, Price, Product Size and Number of Available Soft Drinks in the UK, 2015-19: A Controlled Interrupted Time Series Analysis’, *PLOS Medicine* 17, no. 2 (11 February 2020): e1003025, https://doi.org/10.1371/journal.pmed.1003025.
